# Nuclear export restricts Gdown1 to a mitotic function

**DOI:** 10.1093/nar/gkac015

**Published:** 2022-01-20

**Authors:** Christopher B Ball, Mrutyunjaya Parida, Juan F Santana, Benjamin M Spector, Gustavo A Suarez, David H Price

**Affiliations:** Department of Biochemistry and Molecular Biology, The University of Iowa, Iowa City, IA 52242, USA; Department of Biochemistry and Molecular Biology, The University of Iowa, Iowa City, IA 52242, USA; Department of Biochemistry and Molecular Biology, The University of Iowa, Iowa City, IA 52242, USA; Department of Biochemistry and Molecular Biology, The University of Iowa, Iowa City, IA 52242, USA; Department of Biochemistry and Molecular Biology, The University of Iowa, Iowa City, IA 52242, USA; Department of Biochemistry and Molecular Biology, The University of Iowa, Iowa City, IA 52242, USA

## Abstract

Approximately half of purified mammalian RNA polymerase II (Pol II) is associated with a tightly interacting sub-stoichiometric subunit, Gdown1. Previous studies have established that Gdown1 inhibits transcription initiation through competitive interactions with general transcription factors and blocks the Pol II termination activity of transcription termination factor 2 (TTF2). However, the biological functions of Gdown1 remain poorly understood. Here, we utilized genetic, microscopic, and multi-omics approaches to functionally characterize Gdown1 in three human cell lines. Acute depletion of Gdown1 caused minimal direct effects on transcription. We show that Gdown1 resides predominantly in the cytoplasm of interphase cells, shuttles between the cytoplasm and nucleus, and is regulated by nuclear export. Gdown1 enters the nucleus at the onset of mitosis. Consistently, genetic ablation of Gdown1 is associated with partial de-repression of mitotic transcription, and Gdown1 KO cells present with evidence of aberrant mitoses coupled to p53 pathway activation. Evidence is presented demonstrating that Gdown1 modulates the combined functions of purified productive elongation factors PAF1C, RTF1, SPT6, DSIF and P-TEFb *in vitro*. Collectively, our findings support a model wherein the Pol II-regulatory function of Gdown1 occurs during mitosis and is required for genome integrity.

## INTRODUCTION

RNA Pol II is responsible for the synthesis of all messenger RNAs and various classes of non-coding RNA. Transcription by Pol II is highly regulated to achieve the gene expression profiles distinct to diverse metazoan cell types and accurately respond to environmental stimuli during contexts of stress and developmental signaling. A core machinery consisting of multitudinous protein factors in different macromolecular complexes mediates Pol II initiation at promoters, subsequent promoter-proximal pausing, productive elongation through a chromatinized gene body, and finally, termination, which completes the Pol II transcription cycle (1–4). The set of genes expressed in a given cell type is largely dictated by the chromatin accessibility of promoter elements to the transcriptional machinery, which is established and maintained by transcription factors and chromatin remodeling complexes (5–7). Expression levels are set primarily by positive or negative regulatory transcription factors that are commonly bound within accessible chromatin at promoters and enhancers. The Mediator serves as a conduit of regulatory inputs, bridging the activation function of many distally bound transcription factors to direct transcription pre-initiation complex (PIC) assembly at target gene promoters (8–10). Promoter-proximally paused Pol II may similarly serve as a platform for regulatory inputs directing Pol II entry into productive elongation, as exemplified by the transcriptional response to heat shock (11–13). In addition to gene-specific modes of Pol II transcriptional regulation, global mechanisms of transcriptional control are also known to exist. Examples include transcriptional shutdown during mitosis (14–19), Myc-induced transcriptional amplification (20,21), transcriptional repression pre-zygotic genome activation (22–24), and shutdown of host transcription by certain microbial pathogens (25–27). Indeed, these strategies seem to converge on regulation of core components of the transcriptional machinery.

Gdown1 is a metazoan-restricted, ubiquitously expressed, Pol II-interacting factor that has been described as a sub-stoichiometric Pol II subunit on the basis of its highly stable association with Pol II and the observation that Gdown1 co-purifies with a significant fraction of Pol II prepared from calf thymus and porcine liver (28). In vitro studies of Gdown1 have led to the discovery of several transcription-regulatory properties. It is now firmly established that Gdown1 can block PIC assembly by competitively interfering with the association of Pol II and the general transcription factors TFIIF and TFIIB (29–31). Gdown1 also inhibits the elongation-stimulatory activity of TFIIF, the combined functions of pausing factors DSIF and NELF, and the Pol II termination activity of transcription termination factor 2 (TTF2), with which Gdown1 curiously shares a functionally critical LPDKG motif that is the most conserved motif in both proteins across metazoan evolution (32–35). Further, a partially purified Gdown1 negative accessory factor, GNAF, was shown to cooperate with Gdown1 in vitro to facilitate Pol II pausing (34). These properties potentially qualify Gdown1 as an effector of global transcriptional control, as it would seem that Gdown1 association with Pol II would broadly impact transcription. No specificity factors directing the interaction of Pol II and Gdown1 have been identified. However, the biological functions of Gdown1 have not been extensively explored. Recent studies have established that Gdown1 is essential for the development of *Drosophila* and mice (31,36). Interestingly, one study correlated Gdown1 presence in the nucleus with transcriptional repression in cells of the early *Drosophila* embryo, and noted that Gdown1 shifted to the cytoplasm following or upon zygotic genome activation (31). A separate study carried out in a mouse liver-specific Gdown1 knockout model reported cell cycle defects and a positive functional association of Gdown1 with Pol II productive elongation complexes on a very small number of highly transcribed genes encoding proteins important to liver function (36). However, a means by which Gdown1 is targeted to this specific gene subset was not uncovered.

To better understand the biological functions of Gdown1, we carried out extensive genetic disruption of Gdown1 expression in different human cell types with coupled readouts of gene expression and transcription, characterized the cellular dynamics of Gdown1 localization, and further investigated the in vitro functions of Gdown1 in a reconstituted model of productive elongation. The work presented here suggests that Gdown1 has a minimal direct impact on interphase transcription, which is congruent with a striking discovery that Gdown1 is actively maintained in the cytoplasm of interphase cells. We find that Gdown1 may contribute to mitotic transcriptional repression at the level of initiation and/or elongation and link this function to an observed defect of mitosis in Gdown1 depleted cells.

## MATERIALS AND METHODS

### Cell culture, plasmid and siRNA transfections and drug treatments

HeLa cells and de-identified NHSFs immortalized via retroviral transduction of hTERT (gift of Aloysius Klingelhutz) (37) were grown in Dulbecco's modified Eagle's media (Gibco 11965-092) supplemented with 10% FBS (R&D Systems, S11150). HAP1 cells were grown in Iscove's Modified Dulbecco Media (Gibco 12440-053) supplemented with 10% FBS. All cells were maintained at 37°C and 5% CO_2_. Plasmid transfections were carried out using Lipofectamine 3000 (Invitrogen L3000-008) and siRNA transfections were carried out using Lipofectamine RNAiMax (Invitrogen 13778-150). Double-thymidine blocks were carried out by treating cells with 2 mM thymidine (Millipore Sigma T9250) for 18 h, release into fresh media for 9 h, treatment with 2 mM thymidine for 16 h to uniformly arrest cells at G1/S, and release into S phase with fresh media. Cells were treated with Nocodazole (Millipore Sigma M1404) at 200 ng/ml or DMSO vehicle control at 7 h following release from the second thymidine block and incubated for 5 h to enrich for prometaphase mitotic cells. To study the effects of XPO1/CRM1 inhibition on Gdown1 localization, cells were treated for 4 h with 20 ng/ml Leptomycin B (Millipore Sigma L2913). For PROTAC-mediated degradation of Gdown1, FKBP-Gdown1 cells were treated with 400 nM dTAG^V^-1 (gift of Nathanael Gray) or DMSO vehicle control (final concentration 0.04% v/v) for the indicated times.

### Plasmid design and cloning

A pcDNA 3.1 vector encoding Gdown1 with a single N-terminal FLAG-tag and driven by an HCMV promoter was amplified with primers that directed incorporation of the SV40 NLS at the Gdown1 C-terminus. The linear PCR product was purified with an Invitrogen PureLink Quick Gel Extraction & PCR Purification Combo Kit (K220001), treated with T4 PNK (NEB M0201) to phosphorylate 5′ ends, and subsequently ligated with T4 DNA ligase (NEB M0202) according to manufacturer protocol. The ligated product was transformed into *Escherichia coli* strain DH5α (NEB C2987). Transformed bacteria were incubated in outgrowth media for 1 h at 37°C, spread onto ampicillin selection media, and incubated at 37°C overnight. Colonies were picked up and grown in 3 ml LB supplemented with ampicillin overnight and plasmid DNA was isolated from bacterial cultures via mini-prep (Qiagen 27104). Purified plasmid DNA was submitted for sanger sequencing at the University of Iowa Institute of Human Genetics. See Supplementary Data File for a list of all nucleic acid reagents.

### Single-stranded DNA donor template generation for genome editing

For generation of HA-FKBP-Gdown1 HeLa cells, a gBlock containing, as listed, a left target region homology arm, sequence encoding a Blasticidin (BSD) resistance cassette, P2A ribosomal skipping sequence, two tandem HA tags, FKBP degron domain, and right target region homology arm was designed and ordered from IDT. The design was based on previously described knock-in vectors (38). The gBlock was amplified with primers complementary to the terminal homology arms using KAPA HiFi HotStart ReadyMix (KAPA Biosystems KK2602). One primer used for amplification contained a 5′ phosphate as indicated in Supplementary Data File. The amplified dsDNA product was converted to ssDNA using the TaKaRa Guide-it Long ssDNA Strandase Kit (TaKaRa 632644, original version discontinued – v2 available), which selectively degrades the DNA strand bearing the terminal 5′ phosphate. The ssDNA donor template was compared to the dsDNA starting material via agarose gel electrophoresis and purified using a NucleoSpin Gel and PCR Clean-up kit (Macherey-Nagel, 740609.50). Concentration of the ssDNA product was determined by NanoDrop. The entire preparation of ssDNA donor template (∼5 μg) was ethanol-precipitated and used in a single electroporation reaction for CRISPR-Cas9 mediated knock-in. For eGFP-TEV-Gdown1 HAP1 cells, a gBlock containing a left target region homology arm, eGFP and TEV encoding sequences, and right target homology arm, was ordered from IDT and processed into a donor template as described above.

### CRISPR-Cas9 mediated genome editing and cloning of modified cells

Genome editing was achieved through electroporation of Cas9 RNPs composed of Cas9 and targeting sgRNA consisting of a crRNA: tracrRNA duplex into HeLa or HAP1 cells. In general, for knockouts, 100 pmol of a carrier DNA (IDT 1075915) was included to enhance electroporation. For knock-ins, an ssDNA donor template was included that also served as a carrier DNA and cells were grown in media containing 30 μM Alt-R HDR Enhancer (IDT 1081072), which inhibits non-homologous end-joining, for 48 h following electroporation. Details of Cas9 RNP formation and electroporation are as follows. Custom crRNA (IDT) and Alt-R CRISPR-Cas9 tracrRNA (IDT 1072532) were resuspended at 200 μM concentration in IDTE (IDT 11-05-01-05). The crRNA and tracrRNA were combined at equimolar ratio to form a 100 μM duplex. This combination was heated to 95°C for 5 min, spun down, and allowed to cool to room temperature. Cas9 RNPs were formed by combining 63 pmol of Alt-R S.p. Cas9 Nuclease V3 (IDT 1081058) with 180 pmol crRNA: tracrRNA duplex. This mixture was incubated at 37°C for 10 min and returned to room temperature. The precipitated ssDNA donor template was resuspended in 7 μl IDTE. Approximately 200 000 HeLa or HAP1 cells were resuspended in 20 μl Lonza SE cell line 4-D Nucleofector X Kit S (Lonza V4XC-1032) or Lonza SF cell line 4-D Nucleofector X Kit S (Lonza V4XC-2032) electroporation buffer, respectively. Cells in this buffer were combined with prepared Cas9 RNPs and 100 pmol electroporation enhancer or the complete resuspended ssDNA donor template. This ∼25–30 μl volume was transferred to a Lonza nucleocuvette strip and pulsed in a 4-D Nucleofector X (HeLa code CN-114, HAP1 code EH-100). Afterwards, 75 μl of pre-warmed media was added to the electroporated cells, and the cells were transferred to a 48-well plate for recovery and growth. For knockouts, after 48 h, modified cells were trypsinized, diluted to a theoretical concentration of 2.5 cells/ml, and plated in 200 μl into a 96-well plate. Individual clones were identified, grown, and validated by western blot and PCR. For BSD-P2A-HA-FKBP knock-in, cells were selected with either Blasticidin at 5 μg/ml for 4 days. Surviving cells were given 2 days to recover, and then cloned and characterized as described above. For eGFP-TEV knock-in, modified HAP1 cells were allowed to expand, and eGFP-positive cells detected by FACS on a Becton Dickinson FACS Aria Fusion were individually deposited into single wells of a 96-well plate and allowed to expand for further characterization. For all clones, genomic DNA was isolated using Quick Extract (Lucigen QE0905T) according to manufacturer protocol. PCR screening was carried out with KAPA HiFi HotStart ReadyMix and primers listed in Supplementary Data File. PCR products were analyzed by 1% TAE agarose gel electrophoresis.

### Subcellular fractionation

For separation of the cytosol and stable chromatin-bound/nuclear fraction, HeLa or NHSF cells grown to confluence in a 6-well plate were aspirated of media, washed in ice-cold PBS, and lysed in 200 μl extraction buffer (20 mM HEPES pH 7.6, 150 mM NaCl, 0.5% v/v IGEPAL CA-360, 0.1 mM EDTA, 1 mM DTT, 0.1% isopropanol-saturated PMSF, supplemented with a cOmplete mini protease inhibitor cocktail tablet (Roche 11836153001)). Lysed cells were incubated in the plate on ice for 10 min. Afterwards, cells were scraped and transferred to a 1.5 ml microfuge tube. A sample representing the total cell lysate was reserved, and the remainder was subjected to centrifugation at 13 000 rpm for 10 min at 4°C. The cytosol (supernatant) was separated from the chromatin-bound/nuclear fraction (pellet), and the pellet was resuspended in a volume equal to that of the cytosol. Saponin and Triton extractions were carried out as follows. HeLa cells grown to confluence in a 6-well plate were extracted of soluble proteins with 500 μl of a buffer containing 20 mM HEPES pH 7.6, 150 mM NaCl, 0.05% w/v saponin (Sigma 47036), ±0.5% v/v Triton X-100, 0.1 mM EDTA, 1 mM DTT, 0.1% isopropanol-saturated PMSF and supplemented with a protease inhibitor cocktail tablet. Attached cells were incubated in this buffer for 10 min, and the supernatant was carefully removed. Detached cells/nuclei were removed by centrifugation, and the resulting supernatant was saved for analysis.

### Immunoprecipitation

For IP of GFP-Gdown1 complexes from cells, HAP1 GFP-Gdown1 cells were lysed in a buffer containing 20 mM Tris pH 7.8, 150 mM NaCl, 0.5% (v/v) IGEPAL CA-360, 0.1 mM EDTA, 1 mM DTT, 0.1% isopropanol-saturated PMSF, and supplemented with a cOmplete mini protease inhibitor cocktail tablet. The lysate was clarified of cell debris and chromatin by centrifugation at 13 000 rpm for 10 min at 4°C. A portion of the supernatant was saved as input. GFP-Trap agarose (Chromotek gta-10) was equilibrated in the lysis buffer, and ∼12.5 μl beads were combined with the clarified cell lysate and rotated at 4°C for 1 h. The beads were pelleted and washed in 500 μl lysis buffer three times, and bound proteins were dissociated by incubation in 2× protein sample buffer for 5 min at 95°C. For mass spectrometry, IP of GFP-Gdown1 was carried out as described above, and the beads were washed and resuspended in 50 μl of a buffer containing 20 mM Tris, 100 mM NaCl and 1 mM DTT. Complexes were eluted from the beads via digestion with 50 ng TEV (University of Iowa Protein and Crystallography Facility, TEV-00–001), and the supernatant was submitted to the University of Iowa Proteomics Facility for LC-MS analysis. Data were analyzed using Scaffold 4 software.

### Western blotting

Nuclear pellets and whole-cell lysates generated during this study were supplemented with protein sample buffer and subjected to sonication to shear chromatin prior to gel loading. Protein samples were heated to 95°C for no more than 5 min and analyzed by 10% or 4–20% SDS-PAGE and transferred to nitrocellulose using a semi-dry transfer apparatus. Blots were blocked in 10% milk in 1× PBS with 0.1% Tween for 30 min and then probed with antibodies diluted in 2% milk in PBS with 0.1% Tween. Primary antibody incubations were carried out overnight, except for actin and vinculin blots, which were incubated with primary antibodies for 1 h. HRP-conjugated secondary antibodies were diluted in 2% milk in 1× PBS with 0.1% Tween and incubated for 1 h. Blots were washed 3× for 7 min with PBS-Tween following primary and secondary antibody incubations. Signals were detected using SuperSignal West Femto Maximum Sensitivity Substrate (Thermo Scientific 34095). Data were collected with a UVP Analytik Jena ChemStudio system and analyzed using ImageJ. See Supplementary Data File for a list of all antibodies used in this study and their dilutions.

### Immunofluorescence and EU incorporation assays

Cells were seeded on 12 mm glass coverslips #1.5 (Electron Microscopy Sciences, 50-192-9518) that were coated in rat tail collagen (Corning 354236) and UV-C sterilized. Drug treatments or transfections were carried out as indicated. Cells were aspirated of media and fixed in 4% paraformaldehyde in 1× PBS for 20 min. Afterwards, the cells were washed 3 times in 1× PBS, and then permeabilized with 0.5% Triton X-100 in 1× PBS for 5 min. Cells were washed in 1× PBS twice and then blocked in 10% (w/v) BSA Fraction V (RPI A30075-100.0) prepared in 1× PBS. Cells were then probed with antibodies diluted in 1% BSA in 1× PBS. Primary antibody incubations were carried out at 4°C overnight with gentle rocking. Afterwards, cells were washed 4× for 5 min in 1× PBS and then incubated with Alexa Fluor-conjugated secondary antibodies diluted in 1% BSA in 1× PBS. Cells were then washed 4× for 5 min in 1× PBS, counterstained in Hoescht 33342 diluted to 2 μg/ml in 1× PBS and de-stained for 5 min in 1× PBS. Coverslips were mounted on glass microscope slides (Leica 3800240) in ProLong Diamond Antifade mounting media (Invitrogen P36961), cured at 4°C at least overnight in darkness, and analyzed. For EU incorporation assays, 300 μM EU (Jena CLK-N002-10) was added to the media of live cells for 20 min. Afterwards, crosslinking and permeabilization were carried out as described. EU incorporated into RNA was detected via click chemistry; 250 μl of a click reaction buffer containing 25 mM Tris pH 7.8, 150 mM NaCl, 4 mM CuSO_4_, 20 μM AF 647 Azide (Jena CLK-1299-1) and 100 mM sodium ascorbate was added to the cells and the plate was incubated at room temperature for 1 h in darkness. Afterwards, the reaction buffer was removed and the cells were washed in 1X PBS. Blocking, primary and secondary antibody incubations, counterstaining, and mounting were subsequently carried out as above described. EdU incorporation was carried out using Invitrogen Click-it EdU Cell Proliferation kit for imaging (Invitrogen C10340) according to manufacturer protocol and subsequently stained as above described. For analysis of live GFP-Gdown1 HAP1 cells, cells were seeded on collagen-coated coverslips as described. Cells were removed from media, washed in 1× DPBS with magnesium and calcium chloride (Gibco 14040-133), counterstained with cell permeant dye Hoescht 33342, briefly destained, and inverted onto a microscope slide with a drop of Mg + Ca + DPBS for immediate analysis.

### Microscopy

Microscopy was carried out on a Leica DMR epifluorescent scope fitted with a SPOT RT sCMOS camera and Leica HCX PL APO 63×/1.40–0.60 oil immersion objective. Images were acquired using SPOT software and processed in ImageJ. Features were quantified using the Cell Counter plugin. Fluorescence signals were quantified using the formula: fluorescence = integrated density – (area cell × mean of background measurements). Confocal images of Gdown1 staining in NHSFs were captured on a Zeiss LSM 710 confocal microscope controlled by ZEN software and fitted with a 63X oil immersion objective. This microscope resides at the University of Iowa Central Microscopy Research Facility and was acquired with funds provided by NIH Grant1 S10 RR025439-01. Images of Trypan blue stained asynchronous and mitotic nuclei were collected on a Nikon TMS phase contrast microscope using a Samsung Galaxy S21 and Gosky universal cell phone adapter mount.

### Crystal violet staining and measurements

NHSFs grown in a 6-well plate and transfected with non-targeting or Gdown1 siRNAs for 72 h were aspirated of media and washed once in 1× PBS. Afterwards, the cells were stained with 500 μl 0.5% w/v crystal violet (Sigma C0775) prepared in 20% methanol for 5 min. The crystal violet solution was removed, and the cells were washed twice in 1× PBS and once in water, then allowed to dry overnight. The OD595 of crystal violet stained cells and background control wells was determined using a BioTek Synergy NEO plate reader. A standard curve utilized for data interpolation was generated through serial dilution and staining of NHSFs.

### Nuclei isolation and PRO-Seq

Nuclei isolation was carried out as described previously (39,40), with some modifications made to promote mitotic chromosome retention. For the PRO-Seq experiment that examined transcription in asynchronous and mitotic parental and Gdown1 KO HeLa cells, synchronized prometaphase cells cultured in a T-150 flask were shaken off, poured into a 50 ml conical, pelleted at 500 × g for 5 min, decanted of media, washed in ice-cold PBS, pelleted again, decanted of PBS, and subsequently lysed, rather than being washed and lysed in situ as is standard in our protocol for adherent cell cultures. Asynchronous and mitotic cells were lysed in a buffer containing 20 mM HEPES pH 7.8. 0.1% IGEPAL CA-360, 1 mM EDTA, 0.1% isopropanol-saturated PMSF, 1 mM spermine, 1 mM spermidine, 1 mM DTT, 0.004 U/μl SUPERase-In, 320 mM sucrose, and supplemented with a cOmplete protease-inhibitor cocktail tablet. This lysis buffer contains a reduced amount of IGEPAL CA-360 (normally 1%), which we reasoned may promote the retention of condensed mitotic chromosomes in prometaphase cells that lack a nuclear envelope. Permeabilization was confirmed through Trypan Blue staining of lysed cells. Asynchronous cells were scraped off the flask in lysis buffer and transferred to a conical tube on ice. Lysed cells were spiked in with ∼200 000 Sf21 cells and then pelleted through a 10 mL sucrose cushion containing 20 mM HEPES pH 7.8, 0.1% IGEPAL CA-360, 0.1 mM EDTA, 0.1% isopropanol-saturated PMSF, 1 mM spermine, 1 mM spermidine, 1 mM DTT, 0.004 U/μl SUPERase-In, and 1 M sucrose for 5 min at 22 500 × g. Pellets containing nuclei were then resuspended in 300 μl storage buffer and frozen. PRO-Seq library preparation was carried out as previously described, with the exception that biotinylated nucleotides from Jena Bioscience were utilized (Biotin-11-UTP, Jena NU-821-BIOX, Biotin-11-CTP Jena, NU-831-BIOX, Biotin-11-ATP, Jena NU-957-BIOX-L, Biotin-11-GTP, Jena NU-971-BIOX-L). Libraries were sequenced with 50 bp paired-end reads on an Illumina Hi-Seq 4000 or Illumina Nova-Seq 6000 at the University of Iowa Institute of Human Genetics – Genomics Division.

### RNA-Seq

Total RNA was isolated from cells cultured in a six-well plate using Trizol according to manufacturer instructions. RNA was resuspended in a DNase digestion buffer containing 2 U TURBO DNase (Invitrogen AM2238), its provided reaction buffer, and 20 U SUPERase-in RNase Inhibitor, and incubated at 37°C for 30 min. The reaction was then quenched with the addition of 300 μl Trizol. Afterwards, 300 μl 100% ethanol was added, and the sample was mixed thoroughly. RNA was purified using a Zymo Direct-zol RNA mini-prep kit (Zymo, R0250). The isolated RNA was then submitted for quality control assessment at the University of Iowa Institute of Human Genetics. Only samples with RIN scores >9, as assessed by an Agilent Bioanalyzer 2100, and no significant contamination by DNA or organics, as assessed by a Lunatic Spectrophotometer (Unchained Labs), were retained for analysis. Total RNA-Seq libraries were prepared by the University of Iowa Institute of Human Genetics in biological triplicate using the Illumina TruSeq Stranded Total RNA Library Prep Gold Kit (Illumina 20020598) and sequenced on an Illumina Hi-Seq 4000 with 50 bp paired-end reads.

### ChIP-Seq

ChIP-Seq using the POLR2A and HA antibodies was carried out essentially as described previously (40), with a few modifications. Adherent Parental or HA-FKBP-Gdown1 HeLa cells were crosslinked by the addition of 1% PFA to the media for 10 min. Crosslinking was quenched by the addition of 1 M Tris, pH 7.8, and the cells were scraped, pelleted, washed in ice-cold 1× PBS, pelleted and finally stored at –80°C. Sonication steps were performed using a QSonica Sonicator (30 s on, 30 s off, 30% amplitude, 15 cycles). Samples were pre-cleared with 20 μl Protein A/G PLUS bead slurry (Santa Cruz sc-2003) for 1 h with rotation at 4°C. Afterwards, either 6 μl HA antibody or 4 μg POLR2A antibody were added to the samples, and incubated over night at 4°C with rotation. The following day, samples were combined with 40 μl equilibrated Protein A/G PLUS beads, incubated for 2 h with rotation at 4°C, and then washed, eluted, decrosslinked, and Proteinase K treated as described previously. ChIP DNA was purified using a Qiagen MinElute kit, and libraries were prepared using a NEBNext Ultra II DNA library prep kit (NEB E7645S) using custom Illumina adapters listed in Supplementary Data File. Libraries were sequenced on a NovaSeq 6000 with 50 bp paired-end reads.

### RT qPCR

RNA was extracted with Trizol according to manufacturer instructions. Isolated RNA samples were digested with TURBO DNase (AM2238), then combined with 300 μl Trizol and purified using a ZYMO Direct-zol RNA miniprep kit according to manufacturer instructions. RNA concentration was measured by Nanodrop, and RNA was reverse transcribed into cDNA using SuperScript IV Reverse Transcriptase (Invitrogen 18090010). For qPCR, 5 ng of cDNA was analyzed using Power SYBR Green master mix (Applied Biosystems 4368577) and primers listed in Supplementary Data File on an Applied Biosystems 7900HT instrument at the University of Iowa Institute of Human Genetics.

### FACS

Cells were trypsinized, pelleted, and washed in 1× PBS. A 10X lysis buffer consisting of 10 mM Tris, pH 7.8, 1 mM EDTA, 1% Triton X-100 was prepared. The 10X lysis buffer was diluted to 1× in 10 ml and combined with 10 mg sodium citrate, 25 μl 20 mg/ml propidium iodide (Sigma P4170), and 10 μl RNase A (Thermo Scientific EN0531). Cells were resuspended in 1 ml completed lysis buffer, incubated at room temperature for 30 min, and then analyzed on a Beckton Dickinson LSR II instrument at the University of Iowa Flow Cytometry facility. Data were processed using ModFit.

### *In vitro* transcription

Preinitiation complexes were assembled through incubation of a biotinylated CMV promoter template immobilized to Dynabeads M280 Streptavidin (Invitrogen 11206D) with HeLa nuclear extract for 30 min in 20 mM HEPES pH 7.8, 62.5 mM potassium chloride, 5 mM magnesium chloride, 0.5 U/μl SUPERase-in, 1 mM DTT, and 1 μM Flavopiridol (to eliminate P-TEFb function during the pulse). Transcription was initiated by the addition of a pulse mixture containing 20 mM HEPES pH 7.8, 60 mM potassium chloride, 5 mM magnesium chloride, 1 mM DTT, 1.5 mM AUG mixture (final 0.5 mM each nucleotide), and α-[^32^P]-CTP for 3 min. The pulse was quenched with the addition of 100 μl high salt EDTA wash (HSWE: 20 mM HEPES pH 7.8. 1.6 M potassium chloride, 50 mM EDTA, and 0.02% v/v Tween 20). The beads were pulled down with a magnet, washed twice more with 5 min incubations with HSWE, and then twice with low salt wash (LSW: 20 mM HEPES pH 7.8. 60 mM potassium chloride, and 0.02% v/v Tween 20). The isolated transcription elongation complexes were subjected to a pre-termination protocol that removes remnant TTF2 function through a 10-min incubation in 20 mM HEPES pH 7.8, 60 mM potassium chloride, 5 mM magnesium chloride, 0.5 mM ATP, 1 μM single-stranded oligo, 1 mM DTT, and 0.2 U/μl SUPERase-in. The pre-termination reaction was quenched with the addition of 100 μl low salt EDTA wash (LSWE: 20 mM HEPES pH 7.8, 60 mM KCl, 25 mM EDTA, and 0.02% v/v Tween 20). Beads were washed once more in 200 μl LSWE and twice in 200 μl LSW, and finally resuspended in a sample buffer containing 20 mM HEPES pH 7.8, 60 mM KCl, and 0.2 U/μl SUPERase-in. A sample representing the pulse material was reserved for analysis (lane 1, Figure 5A, B). A fraction of the elongation complexes was then incubated with 30 pmol Gdown1 (34) for 10 min. Excess Gdown1 was washed away with two washes in 200 μl LSW, and the beads were resuspended in sample buffer. Elongation complexes with and without associated Gdown1 were divided into 9 ul aliquots, and addbacks of the indicated amounts purified PAF1C, Spt6, Rtf1, DSIF and P-TEFb (2,41) diluted in sample buffer were carried out (20 μl final volume for each sample). Samples were incubated for 10 min, and then subjected to a 3-min chase with the addition of 4 μl of a chase mix containing 20 mM HEPES pH 7.8, 60 mM potassium chloride, 18 mM magnesium chloride (final 3 mM), 1 mM DTT, and 3 mM AUGC mix (final 0.5 mM each nucleotide). Reactions were quenched with the addition of 200 μl Torula yeast stop solution (100 mM Tris pH 7.6, 0.2 mg/ml Torula yeast RNA, 20 mM EDTA, 1% Sarkosyl). Nucleic acids were isolated via phenol extraction and ethanol-precipitated. Samples were resuspended in 10 μl RNA loading buffer and separated on by 6% urea–TBE PAGE. The gel was stained with ethidium bromide to visualize template DNA, dried, and exposed to a phosphor screen. Signals were detected using a GE Typhoon FLA 7000 instrument and data were processed using FUJI Multigauge software.

### Bioinformatics

PRO-Seq data were worked up using an automated python pipeline called RNAfastqtoBigWig. The program downloaded raw fastq.gz data from the University of Iowa Institute of Human Genetics server, trimmed Illumina adapter sequences using trimGalore and aligned to a combined hg38 and *Spodoptera frugiperda* genome file (WGS number JQCY02.1) using the bowtie aligner. Next, the program collapsed identical mapped reads with redundant UMIs and removed the biotinylated NTP from the 3′ end using the dedup program, converted the resulting bed file into bedGraphs using the bedtools genomecov program, corrected for library depths considering total and spike-in reads across samples as previously described (39), and generated bigWig tracks using the bedGraphToBigWig program. Signals across biological duplicates were summed using the bigWigCompare program. Pause regions and gene body annotations at transcribed genes were generated for our HeLa NasCap data using the truQuant algorithm (42). Genomic coverage of pause and gene body regions for Parental and Gdown1 KO #3 PRO-Seq datasets were quantified using the bedtools coverage program and compared using the DESeq2 program. The tsrFinderM1 algorithm was applied to our HeLa NasCap data (43) with default parameters to discover TSRs and their respective MaxTSSs. The bedtools coverage program was used to calculate total fragments in 500 bp genomic intervals centered on MaxTSSs from our HeLa H3K4me1 and H3K4me3 datasets (40). Enhancers were defined as genomic intervals with a signal ratio of H3K4me1 over H3K4me3 >1. The ratio of Parental HeLa and Gdown1 KO #3 PRO-Seq 5′ end signals in the 500 bp genomic intervals were quantified using the bedtools coverage program and utilized for data sorting.

RNA-Seq datasets were downloaded and trimmed of Illumina adapters and polyA tails using trimGalore. Data were aligned to the hg38 genome assembly using hisat2 and converted to bed files using the bedtools bamtobed split function to account for splicing. An awk script was used to maintain pairwise orientation of reads according to the strand of the first mate in a pair of mapped reads. The bedtools genome coverage program was used to generate bedGraph from bed files and the bedGraphToBigWig program was used to convert them into bigWig files. The Featurecounts program was used to quantify total number of strand specific fragments in all the transcripts from the GENCODE Release 38 (GRCh38.p13) basic gene annotation. The DESeq2 program was used to analyze these quantifications for differential gene expression analysis. DESeq2 scaling factors were applied to bedGraph files prior to their conversion to bigWigs, which are presented in figures as the summed signals of three biological replicates.

ChIP-Seq datasets were processed using the DNAfastqtoBigWig program that, as for PRO-Seq, trims, aligns, and deduplicates the sequencing reads and converts the aligned data into bigWigs. The Pol II and HA ChIP-Seq datasets were normalized to total reads. Tracks subtractions were generated using the bigwigCompare program with the –binSize 500 and –skipZeroOverZero options. Metaplots of normalized Pol II and HA ChIP-Seq signals were generated for regions ±1 kb from the MaxTSS of defined truQuant genes using bedtools coverage and a custom awk script.

Links to code for the bioinformatics programs utilized are listed in Table 1.

**Table 1.** Code availability

Program	Link
trim_Galore	https://github.com/FelixKrueger/TrimGalore/releases/tag/0.6.6
bowtie	http://bowtie-bio.sourceforge.net/index.shtml v1.2.2
hisat2	http://daehwankimlab.github.io/hisat2/
dedup	https://github.com/P-TEFb/dedup
RNAfastqtoBigWig	https://github.com/P-TEFb/RNAfastqtoBigWig
DNAfastqtoBigWig	https://github.com/P-TEFb/DNAfastqtoBigWig
bigwigCompare	https://deeptools.readthedocs.io/en/develop/content/tools/bigwigCompare.html
featureCounts	https://www.rdocumentation.org/packages/Rsubread/versions/1.22.2/topics/featureCounts
DESeq2	https://bioconductor.org/packages/release/bioc/html/DESeq2.html
tsrFinder	https://github.com/P-TEFb/tsrFinderM1
bedGraphToBigWig	https://www.encodeproject.org/software/bedgraphtobigwig/
Bedtools	https://bedtools.readthedocs.io/en/latest/index.html
truQuant	https://github.com/meierjl/truQuant

### Statistics

Paired and unpaired two-tailed Student's *t*-tests were carried out in Microsoft Excel or GraphPad Prism. DESeq2 measured the significance of gene expression changes in RNA-Seq and PRO-Seq datasets, and for all presented analyses, adjusted *P*-values were utilized.

## RESULTS

### Loss of Gdown1 activates the p53 pathway and is associated with aberrant mitoses

**Figure 1.**
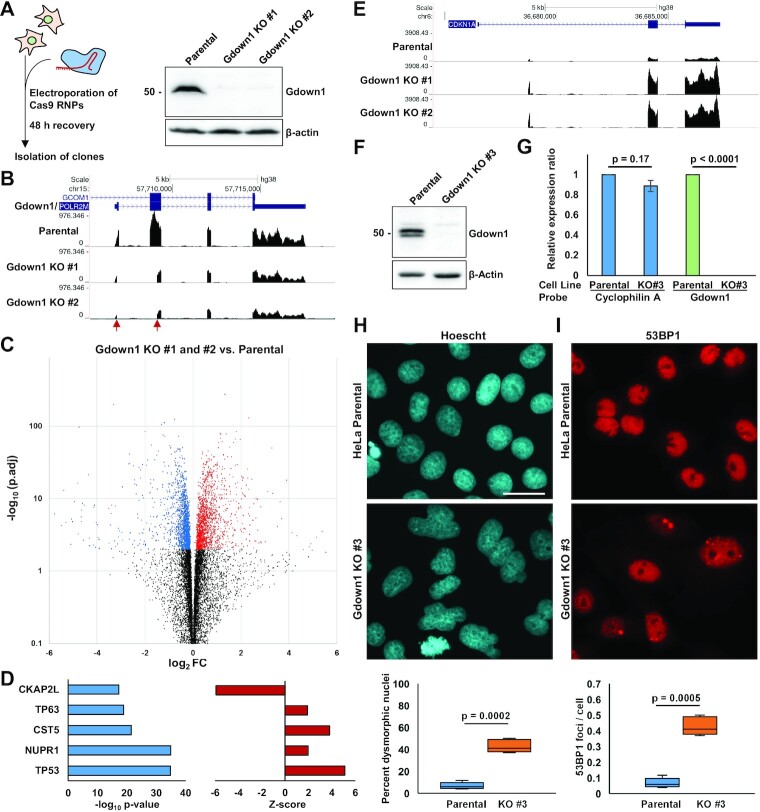
Loss of Gdown1 in HeLa cells is associated with p53 pathway activation and aberrant mitoses. (**A**) Left: Schematic for generation of Gdown1 KO clones. Pre-formed Cas9 RNPs containing guide RNAs that target the Gdown1 locus were electroporated into HeLa cells. Cells were given 48 h to recover, and then plated as individual clones for propagation and analysis. Right: Western blot analysis of two isolated Gdown1 KO clones, named Gdown1 KO #1 and KO #2. (**B**) UCSC genome browser snapshot of total RNA-Seq coverage over the Gdown1 locus in parental and Gdown1 KO cells. Red arrows indicate the location of Cas9 cut sites. (**C**) Volcano plot analysis of differentially expressed genes (DEGs) in KO #1 and KO #2 HeLa cells relative to the Parental cell line. DEGs that are downregulated and upregulated with an adjusted p-value of less than 0.01 are colored in blue and red, respectively. (**D**) Qiagen Ingenuity Pathway Analysis of DEGs colored in (C), with p. adj. < 0.01, indicating top upstream regulators of DEGs ranked by –log_10_ (p. adj.) of gene set overlap in left panel. *Z*-scores for each regulator are shown in the right panel. (**E**) UCSC genome browser snapshot of total RNA-Seq data showing upregulation of CDKN1A / p21, a canonical p53 target gene, in Gdown1 KO #1 and #2 HeLa cells compared to the parental cell line. (**F**) Western blot showing loss of Gdown1 protein in Gdown1 KO #3 HeLa cells. (**G**) RT-qPCR analysis of Cyclophilin A and Gdown1 mRNA levels in parental and Gdown1 KO #3 HeLa cells. Relative expression was determined using the ΔΔ Ct method using the 18S ribosomal rRNA as a reference probe. Data were averaged and normalized to parental cell line values (*n* = 3 biological replicates, with two technical replicates; significance was determined using a paired Student's *t*-test). (**H**) Top: Hoescht 33342 staining of Parental HeLa cells and Gdown1 KO #3 HeLa cells, showing evidence of dysmorphic nuclei in the knockout cell line. Scale bar = 50 μm. Bottom: Box-plot quantification of percentage of dysmorphic nuclei in 5 separate fields (significance determined using paired Student's *t*-test). (**I**) 53BP1 staining of Parental and Gdown1 KO #3 cells, showing an increased number of 53BP1 foci marking DNA double-strand breaks. Scale bar = 50 μm. Bottom: Box-plot quantification of 53BP1 foci/cell in five separate fields (significance determined using paired Student's *t*-test).

As a preliminary approach to characterizing the biological function of Gdown1, Gdown1 knockout HeLa cell lines were created by CRISPR-Cas9 mediated deletion of a 2.3 kb region spanning Gdown1 exons 1 and 2 that encodes the N-terminal Gdown1 amino acids 4–174. This deleted region includes the highly conserved LPDKG motif, which is required for stable association of Gdown1 with isolated Pol II elongation complexes (32), and most of a characterized N-terminal region (amino acids 1–67) that stabilizes the Gdown1-Pol II interaction and strongly contributes to initiation inhibition (31). Two viable clones (KO #1 and #2) with deletion of this region were isolated and produced no Gdown1 protein by western blot, indicating that Gdown1 is non-essential in HeLa cells (Figure 1A, Supplementary Figure S1A). In keeping with this result, a recent study found that while Gdown1 was essential for murine development, liver-specific Gdown1 KO mice were viable and liver cell function was apparently not acutely compromised (36). To determine whether loss of Gdown1 was associated with specific changes in gene expression, total RNA from parental HeLa cells and both knockout clones was analyzed by sequencing in biological triplicate. RNA-Seq coverage over the Gdown1 locus revealed that mRNA produced in the two knockout clones precisely lacked exonic coverage between the two Cas9 target sites (indicated by red arrows) (Figure 1B). Although it is possible that these mRNAs yield a truncated Gdown1 protein that is not recognized by our antibody, it would likely be highly defective given the deletion of the aforementioned Pol II control region. Differential gene expression analysis carried out with DESeq2 (44) revealed only a modest overlap of gene expression differences in the two knockout clones compared with the parental cell line, with approximately 40 percent of significantly up- and downregulated genes (*P*_adj._ < 0.01) shared between them (Supplementary Figure S1C). DESeq2 was therefore leveraged to find genes that were differentially expressed in the same direction in both knockout clones, presented as a volcano plot in Figure 1C (1646 up, 1533 down, *P*_adj._ < 0.01, *n* = 13 429 expressed transcripts). Qiagen Ingenuity Pathway Analysis of these differentially expressed genes (DEGS) identified p53 as the top upstream effector of observed gene expression changes based on target gene overlap and *Z*-score analysis was congruent with p53 being activated (Figure 1D). Indeed, CDKN1A / p21, a direct target of p53 transactivation, was significantly upregulated in both knockout clones, as were many other direct p53 targets (45) and indirectly repressed gene targets associated with cell cycle and G2/M progression (46) (Figure 1D, E, Supplementary Figure S1D).

To rule out the possibility that a truncated, partially functional Gdown1 protein might impact our conclusions, a third HeLa Gdown1 knockout clone (KO #3) was generated in which the entire Gdown1 coding region was deleted. These cells were viable and produced no detectable Gdown1 protein by western blot, nor any Gdown1 mRNA by RT-qPCR (Figure 1F, G and Supplementary Figure S1B). Visual inspection gave evidence of large, dysmorphic nuclei and incomplete mitoses with a significantly increased occurrence of DNA double-strand breaks as detected by 53BP1 foci (Figure 1H, I). Notably, these findings are in agreement with recent observations that Gdown1 ablation in mouse liver is associated with incomplete mitoses and p53 pathway activation (36).

To extend our investigation to other cell types, Gdown1 was knocked out in HAP1 cells, a chronic myelogenous leukemia derivative that is near-haploid. Five viable clones in which the entire Gdown1 coding region was deleted were isolated, and microscopic analysis further supported the finding that loss of Gdown1 is associated with aberrant mitoses, as evidenced by an increase in the number of chromosome bridges in anaphase and telophase mitoses, which were observed in approximately 16% of parental HAP1 cells and 42% of Gdown1 KO HAP1 cells (Supplementary Figure S1E, F). In addition, an siRNA was used to knock down Gdown1 in TERT-immortalized normal human skin fibroblasts (NHSFs). Gdown1 knockdown was associated with an induction of p21 protein expression, a significant, nearly 2-fold reduction in cell growth or viability over a period of 72 h, and a modest, yet significant increase in G1/G0 phase cells with a corresponding decrease in S and G2/M phase cells (Supplementary Figure S1G−I). These observations in NHSFs are collectively supportive of p53 pathway activation and possibly cell cycle arrest or cell death following mitosis as a result of loss of Gdown1 function.

Of note, both HeLa cells and HAP1 cells exhibit only partial p53 function, which may be linked to their survival following Gdown1 loss, whereas p53 signaling is completely intact in NHSFs, possibly highlighting the apparent cell death and growth arrest phenotype following Gdown1 knockdown (47,48). Taken together, our results establish that under standard growth conditions, Gdown1 is non-essential in HeLa and HAP1 cells. Gdown1 deficiency is primarily associated with a defect in cell cycle progression, possibly during mitosis, which leads to p53 pathway activation.

### Gdown1 minimally impacts interphase transcription

**Figure 2.**
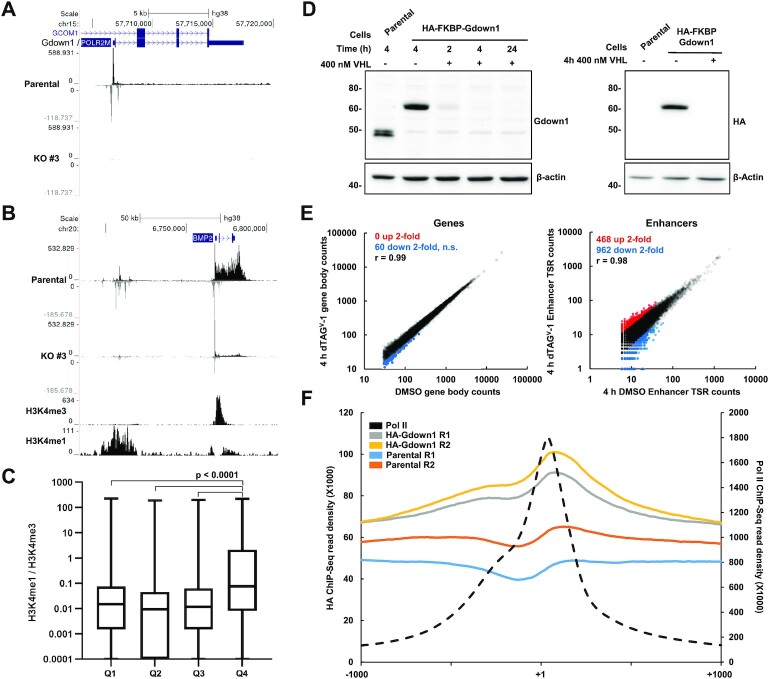
Effects of Gdown1 KO and acute depletion on Pol II transcription. (**A**) PRO-Seq coverage of the Gdown1 locus in Parental and Gdown1 KO #3 HeLa cells, showing complete loss of signal in the KO #3 cell line. (**B**) Genome browser snapshot of highly transcribed BMP2 gene and upstream enhancers that are transcriptionally downregulated in Gdown1 KO #3 HeLa cells. (**C**) Boxplot quantification of H3K4me1 / H3K4me3 ratio across TSRs sorted as in Supplementary Figure S2F, split into quartiles. Significance determined by paired Student's *t*-test. (**D**) Western blot analysis of Parental and HA-FKBP Gdown1 cells treated with vehicle control or 400 nM VHL for the indicated times. Blots were probed to detect both the endogenous and HA-tagged Gdown1 proteins. (**E**) Left: Correlations of PRO-Seq truQuant gene body counts in HA-FKBP-Gdown1 cells treated for 4 h with DMSO vehicle control or 400 nM dTAG^V^-1. No statistically significant differences (*P*_adj._ < 0.05) in transcription of gene bodies were detected through pairwise comparison of gene body counts in DESeq2. *r* = Pearson's correlation coefficient. Right: Correlation of PRO-Seq 5′ end counts in putative active enhancer TSRs. (**F**) Metaplots of Pol II ChIP signal from Parental HeLa cells and HA ChIP signals from Parental and HA-FKBP-Gdown1 cells at truQuant gene promoters (*n* = 12 291) −/+ 1 kb. Note that the Pol II ChIP signal is presented with a different scale.

RNA-Seq reports primarily on the abundance of long, mature RNAs, and thus is not informative on effects at individual stages of the Pol II transcription cycle (e.g. promoter-proximal pausing, productive elongation), nor does it robustly measure unstable Pol II transcripts such as enhancer RNAs. To better ascertain how loss of Gdown1 is associated with altered Pol II transcription, spike-in controlled PRO-Seq, which quantitatively measures nascent Pol II transcripts (49), was performed in parental and Gdown1 KO #3 HeLa cells. Libraries were prepared in biological duplicate and comparison of gene body counts between replicates indicated a very high degree of correlation (Supplementary Figure S2A). As expected, PRO-Seq coverage of the Gdown1 locus was eliminated in KO #3 cells (Figure 2A). DESeq2 was utilized for pair-wise comparison of gene body counts (n = 12,291 transcribed genes) between parental and KO #3 cells, and revealed a surprisingly low number of genes that exhibited significant differential transcription (489 up, 178 down, *P*_adj._ < 0.01) (Supplementary Figure S2B). In agreement with observations from RNA-Seq analyses, transcriptionally upregulated genes included many p53 targets, such as CDKN1A/p21, and p53 was suggested to be activated by pathway analysis (Supplementary Figure S2C, D). A recent study observed that Gdown1 loss in mouse liver is associated with reduced Pol II occupancy over a small number of highly transcribed genes that play critical roles in liver metabolism, and that this reduction was mechanistically linked to Gdown1 occupancy within the bodies of these genes (36). Among the most significantly downregulated genes in our data were CPS1 and ASS1, which interestingly were identified as targets negatively regulated by Gdown1 knockout in the mouse liver study (Supplementary Figure S2B). Of note, transcription of these genes in HeLa cells is similarly characterized by a very high level of productive elongation.

Further examination of the PRO-Seq data suggested that transcription of certain enhancers, enriched for H3K4me1 as compared to H3K4me3, was impacted in Gdown1 KO #3 cells. This is exemplified by the enhancer cluster upstream of the highly transcribed and downregulated BMP2 gene (Figure 2B). To study these effects globally, actively transcribed enhancers were identified by defining 20 bp transcription start regions (TSRs) from previously published HeLa NasCap data, and quantified H3K4me1 and H3K4me3 ChIP-Seq signals within a 500 bp window surrounding the major transcription start site (TSS) of each TSR. H3K4me3 is a histone modification enriched at active gene promoters, whereas monomethylated H3K4 is enriched at enhancers. TSRs associated with a H3K4me1 / H3K4me3 ratio greater than 1 were defined as putative active enhancers. In total, 174 098 TSRs were identified, of which 22 634 were defined as enhancers (Supplementary Data File). A genome browser track documenting all TSRs (black) and the subset defined as enhancers (green) is shown for the downregulated CUX1 gene, which harbors many downregulated enhancers within its long introns (Supplementary Figure S2E). Quantification of PRO-Seq 5′ ends within all TSRs in the parental and Gdown1 KO #3 PRO-Seq data, sorted by the ratio of KO #3 to Parental counts, reveals that significantly downregulated TSRs are enriched for putative active enhancers, although clearly some enhancers exhibited increased levels of transcription, and our initial annotation of enhancers using HeLa NasCap data does not consider TSRs that are upregulated above background in Gdown1 KO cells (Figure 2C, Supplementary Figure S2F).

To ascertain the initial direct effects of Gdown1 removal, a HeLa cell line was generated in which Gdown1 was N-terminally tagged at each endogenous locus with two HA epitopes and an FKBP^F36V^ domain, enabling rapid PROTAC-mediated degradation (50). This strategy minimizes secondary effects downstream of factor removal. The engineering of these cells was validated by PCR and western blot, and the expected ∼15 kDa size increase in the Gdown1 protein was readily apparent (Figure 2D, Supplementary Figure S2G). HA-FKBP-Gdown1 appears to function normally, as it largely co-fractionated with Pol II by glycerol gradient sedimentation and mirrored the fractionation pattern of WT Gdown1 (Supplementary Figure S2H). Treatment of cells with 400 nM dTAG^V^-1 (50) (a gift of the Nathanael Gray lab) depleted Gdown1 to virtually undetectable levels within 4 h (Figure 2D). PRO-Seq analysis of Parental and HA-FKBP-Gdown1 HeLa cells following 4 h treatment with 400 nM dTAG^V^-1 or DMSO was performed. Duplicate libraries were characterized by a high level of correlation in pause region and gene body counts, and it was not apparent that dTAG^V^-1 treatment of parental cells had any significant impact on transcription (Supplementary Figure S2I, J). Remarkably, acute depletion of Gdown1 was not associated with any statistically significant changes in the transcription of 12 291 genes or 22 634 pre-defined enhancers, suggesting that Gdown1 does not play a major direct role in global Pol II transcription (Figure 2E and Supplementary Data File).

To test whether Gdown1 exhibits potentially functional occupancy on chromatin, ChIP-Seq for Pol II and Gdown1 was carried out, leveraging use of a specific HA antibody for ChIP of Gdown1. As a background control, the HA antibody was utilized to IP chromatin from the unmodified parental HeLa cell line. Pol II ChIP-Seq signals exhibited standard patterns of pausing and productive elongation at actively transcribed genes, whereas HA-FKBP-Gdown1 ChIP signals were weakly enriched relative to the background control at genic pause regions, suggestive of a very limited, but perhaps functional association of Gdown1 with paused Pol II (Figure 2F). Taken together, our data suggest that Gdown1 does not play a major functional role in most interphase Pol II transcription in HeLa cells and, correspondingly, exhibits very limited engagement of chromatin. Although there are pronounced effects on transcription and gene expression in Gdown1 KO cells, these may be secondary, a unique feature of the isolated clone, or require more than 4 h to manifest following acute Gdown1 removal.

### Gdown1 is predominantly cytoplasmic and its localization is controlled by nuclear export

**Figure 3.**
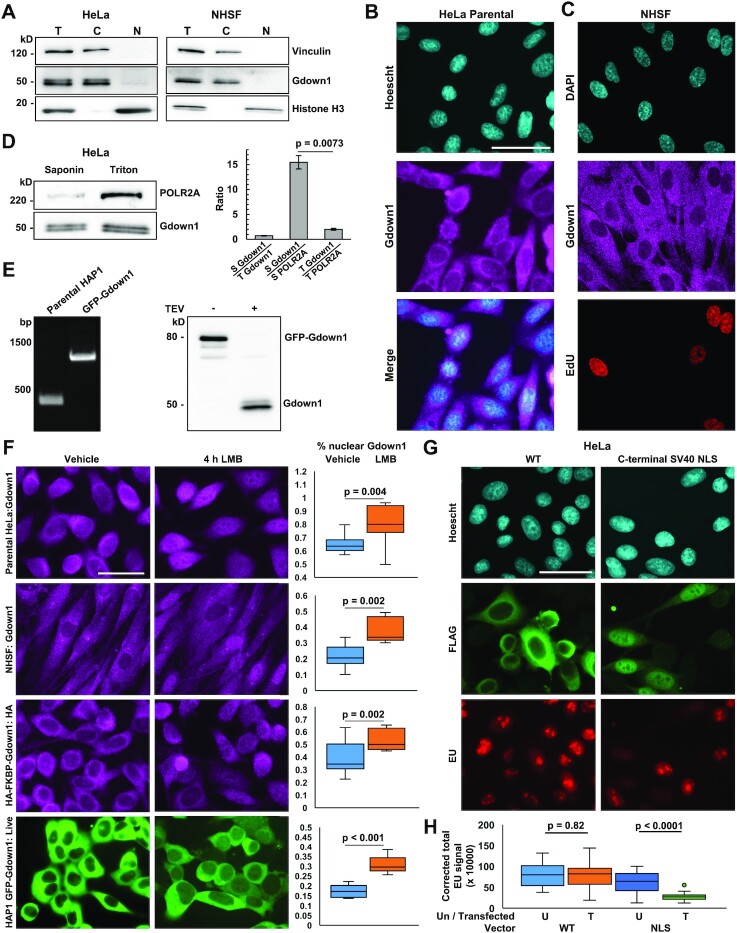
Gdown1 is largely not associated with chromatin, localizes predominantly to the cytoplasm, and is regulated by nuclear export in three human cell types. (**A**) Western blot analysis of Vinculin (cytoplasmic), Gdown1, and Histone H3 (chromatin-associated) in total (T), cytosolic (C), and nuclear (N) fractions derived from HeLa cells (left) and NHSFs (right). (**B**) Indirect immunofluorescence detecting Gdown1 in Parental HeLa cells. Scale bar = 10 μm. (**C**) Confocal images of Gdown1-stained NHSFs probed for EdU to mark S-phase cells. (**D**) Left: western blot analysis of Gdown1 and POLR2A in the soluble fraction of saponin and Triton-extracted HeLa cells. Right: Quantification of Gdown1 signal in saponin extracted cells / Gdown1 signal in Triton extracted cells and Gdown1 / Pol II signals in saponin and Triton extracted cells. Significance determined by unpaired Student's *t*-test. (**E**) Left: PCR validation of eGFP-TEV-encoding sequence insertion at the Gdown1 N-terminus in HAP1 cells. Right: Western blot of native lysates prepared from eGFP-TEV-Gdown1 HAP1 cells treated with TEV or untreated. Blot probed with Gdown1 antibody. (**F**) Fluorescence microscopy revealing predominantly cytoplasmic localization of Gdown1 in Parental HeLa cells, NHSFs, HA-FKBP-Gdown1 HeLa cells and eGFP-TEV-Gdown1 HAP1 cells, and evidence accumulation in the nucleus after 4 h treatment with the XPO1 / CRM1 inhibitor Leptomycin B at 20 ng/ml. Antibodies used for indirect immunofluorescence are indicated after the cell line identifier. eGFP-TEV-Gdown1 was visualized in live cells (see methods for details). Scale bar = 50 μm. Quantification of the fraction of Gdown1 in the nucleus in control and Leptomycin-treated cells is at the right. (**G**) Indirect immunofluorescence visualizing the localization of exogenously expressed WT FLAG-Gdown1 and FLAG-Gdown1-SV40 NLS in HeLa cells, and effect of their expression on global transcription measured by EU incorporation and click-chemistry attachment of AlexaFluor 647 dye. Scale bar = 50 μm. (**H**) Boxplot quantification of corrected total EU signal in untransfected cells and cells transfected with WT FLAG-Gdown1 or FLAG-Gdown1-SV40 NLS. Untransfected cells within each transfection experiment were quantified to control for variation in EU incorporation efficiency and click-chemistry efficiency across conditions. Untransfected = U, Transfected = T. The transfected vector is indicated along the bottom of the plot. Significance was determined using a paired Student's *t*-test.

The observation that Gdown1 exhibited limited association with chromatin was surprising, as previous studies have documented that virtually all Gdown1 is associated with Pol II, which is consistent with our glycerol gradient results, and reported Gdown1 occupancy variously over TSSs (29,34), sites of promoter-proximal pausing (34), and within a limited number of target gene bodies (36). However, the strongly inhibitory function of Gdown1 on Pol II preinitiation complex assembly draws into question whether or how Gdown1 is associated with initiating Pol II, and how Gdown1 might be retained through the elongation phase of the transcription cycle. To bolster the observation that Gdown1 is largely not associated with transcribing Pol II on chromatin, subcellular fractionation of HeLa cells and NHSFs was performed. Remarkably, almost all Gdown1 was found in the cytosolic fraction of both cell types by western blot (Figure 3A).

A recent study reported that Gdown1 is found in the cytoplasm of certain cells in the developing *Drosophila* embryo (31). Therefore, we examined Gdown1 localization in HeLa cells by indirect immunofluorescence with a Gdown1 antibody, and indeed observed strong signals in the cytoplasm and an apparent fringe of high signal at the edge of the nucleus (Figure 3B). A similar result was observed via confocal microscopic analysis of NHSFs stained for Gdown1 (Figure 3C). Gdown1 was detected primarily in the cytoplasm of EdU labeled S-phase cells and EdU-negative cells, suggesting that Gdown1 is predominantly cytoplasmic during all of interphase and that the aberrant mitotic phenotype of Gdown1-depleted cells is unlikely to be related a transcription-regulatory function of Gdown1 during genome replication. To further characterize Gdown1 subcellular distribution, saponin and Triton extractions of HeLa cells were carried out. Saponin preferentially permeabilizes the plasma membrane, allowing for the release of soluble cytoplasmic proteins in a gentle condition, whereas Triton solubilizes all cellular membranes and allows nucleoplasmic proteins to be efficiently released as well. Notably, saponin and Triton extraction recovered similar levels of Gdown1, whereas soluble Pol II was significantly more abundant in the Triton extracted sample (Figure 3D). These results collectively suggest that Gdown1 and Pol II exist predominantly in separate cellular compartments and apparently contradict previous observations that all Gdown1 is associated with Pol II, which is almost entirely nuclear. These results can be reconciled as standard lysis protocols rely on cellular decompartmentalization. Under such conditions it is likely that Gdown1 would be quickly bound to Pol II, given the extraordinarily high stability of their interaction. Previous studies suggesting that the majority of Gdown1 is associated with Pol II based this conclusion on the purification and chromatographic separation of Pol II and Pol II: Gdown1 from calf thymus and porcine liver lysates (28). To our knowledge, the subcellular distribution of Gdown1 in these tissues, as assessed by microscopy, has not been reported, and would be of interest to query given our present findings.

To investigate further, an eGFP-TEV-Gdown1 cell line was generated by CRISPR-Cas9 modification of the endogenous Gdown1 locus in HAP1 cells. PCR and western blotting confirmed the appropriate modifications, and TEV digestion of a native cell lysate recovered Gdown1 that typically migrates at 50 kDa and is characterized by a doublet resulting from different phosphorylation states (32) (Figure 3E). We hypothesized that the fringe of Gdown1 signal around the nucleus may reflect Gdown1 shuttling across the nuclear envelope. The localization of Gdown1 in parental HeLa cells, NHSFs, HA-FKBP-Gdown1 HeLa cells, and live eGFP-TEV-Gdown1 HAP1 cells following 4 h treatment with the nuclear XPO1/CRM1 inhibitor Leptomycin B or vehicle control (antibodies indicated) was therefore assayed. Congruent with preliminary observations, Gdown1 was observed to be primarily cytoplasmic in all cell types under control conditions (Figure 3F). Strikingly, a significant fraction of Gdown1 shifted to the nucleus upon XPO1 inhibition, indicating that Gdown1 is indeed regulated by nuclear export. In support of this idea, it was observed through the use of NES detection algorithms that Gdown1 contains multiple candidate XPO1 nuclear export signals (data not shown). Of note, no canonical nuclear localization signals were identified in the Gdown1 protein.

To test how forced direction of Gdown1 to the nucleus impacts transcription, transfection vectors expressing either WT FLAG-Gdown1 or FLAG-Gdown1 fused to a strong C-terminal SV40 nuclear localization signal (NLS) were developed. HeLa cells were transfected for 24 h and Gdown1 localization was assayed by immunofluorescence using the FLAG-antibody. Transcriptional effects associated with expression of each Gdown1 construct were simultaneously assayed by fluorescent EU incorporation assay (Figure 3G). As expected, WT FLAG-Gdown1 was found almost exclusively in the cytoplasm. Interestingly, the SV40 NLS strongly shifted the balance of FLAG-Gdown1 to the nucleus. Expression of WT Gdown1 had no effect on EU incorporation compared to untransfected cells, while expression of the NLS fusion strongly reduced EU incorporation in the nucleus, consistent with Gdown1 globally blocking initiation (Figure 3H). Interestingly, bright puncta of EU signals corresponding to Pol I transcription in nucleoli were also strongly reduced in FLAG-Gdown1-NLS transfected cells, suggesting a potential coupling of Pol II and Pol I transcriptional activity. Alternatively, over-expressed Gdown1 that is driven to the nucleus may functionally interact with Pol I, Pol II, and Pol III through shared polymerase subunits (29).

In consideration of potentially significant cytoplasmic interactions, mass spectrometric analysis of eGFP-TEV-Gdown1 immunoprecipitates from HAP1 cells was performed. Only those proteins specifically eluted by TEV digestion were analyzed. Interestingly, in addition to Pol II subunits, Gdown1 enriched for RPAP2 and GPN3, two factors with documented roles in Pol II biogenesis and import (53–56) that also show partial cytoplasmic localization and shuttle between the nucleus and cytoplasm (Supplementary Figure S3A). There was also a partial enrichment for the subset of Pol II subunits thought to be contained within an RPB2/POLR2B and RPB3/POLR2C-containing sub-assembly formed in the cytoplasm during Pol II biogenesis (51,52). These results were corroborated by silver staining and RPAP2 western blot analysis of eGFP-Gdown1 immunoprecipitates (Supplementary Figure S3A−C). It was therefore tested whether loss of Gdown1 was associated with a Pol II biogenesis or import defect, which has previously been associated with cytoplasmic accumulation of Pol II subunits (54,56). Most of the Pol II was still found in the nucleus after depletion of Gdown1, but there may have been a slight cytoplasmic accumulation of POLR2A (Supplementary Figure S3D). In agreement with our observation that Gdown1 loss is associated with defective mitoses, cells depleted of Gdown1 for 24 h, enough time for most HeLa cells to complete a cell cycle, exhibited moderately dysmorphic nuclei (Supplementary Figure S3D). The relatively subtle phenotype of cells acutely depleted of Gdown1 in comparison to the knockout may be attributed to the knockout cells having gone through many cell divisions in the absence of Gdown1 during their isolation. Additionally, levels of cellular Pol II were not substantially altered following 24 h of Gdown1 depletion (Supplementary Figure S3E). Of note, RPAP2 was also recently shown to interfere with transcription initiation in vitro, and its position in a binary complex with Pol II is sterically incompatible with transcription (53). Taken together, our results establish that Gdown1 is primarily cytoplasmic in three distinct human cell types, not associated with the vast majority of transcriptionally active Pol II, and is retained in the cytoplasm during interphase by mechanisms involving nuclear export. Forced direction of Gdown1 to the nucleus strongly interfered with global transcription, presumably at the level of initiation.

### Gdown1 may repress mitotic transcription

**Figure 4.**
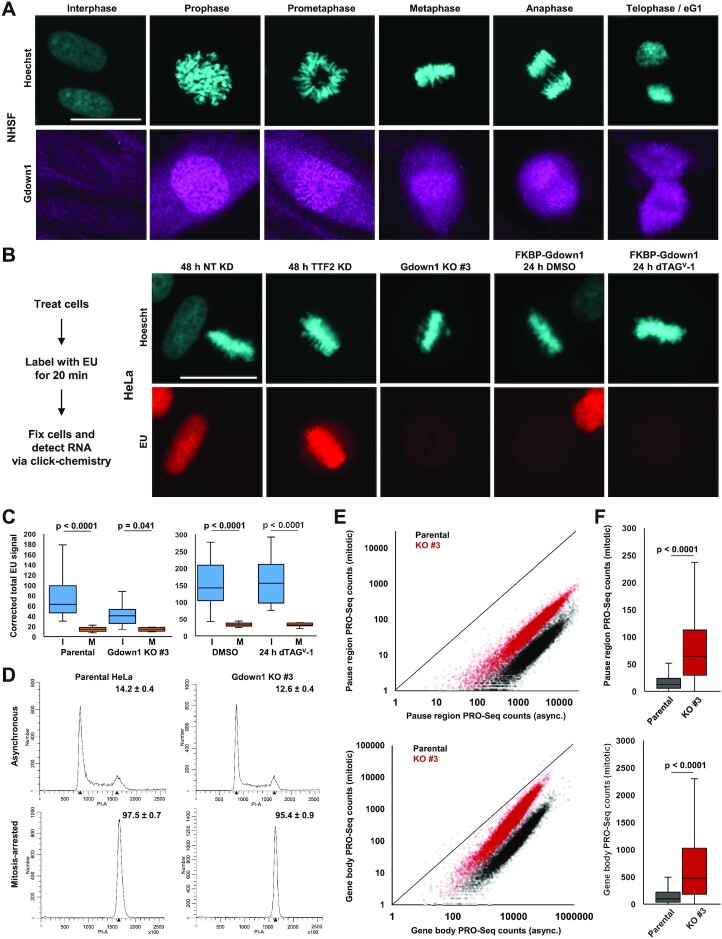
Gdown1 may repress mitotic transcription. (**A**) Indirect immunofluorescence of Gdown1 in NHSFs during interphase and the indicated mitotic phases (Prophase, Prometaphase, Metaphase, Anaphase and Telophase/early G1 (eG1)). Scale bar = 25 μm. (**B**) Left: schema for EU incorporation assays. Right: representative images of EU incorporation assay measuring transcriptional activity in parental HeLa and Gdown1 KO #3 mitotic cells, parental HeLa cells depleted of TTF2 by siRNA interference for 48 h (parental control is NT KD), and HA-FKBP-Gdown1 mitotic cells treated with DMSO vehicle control or 400 nM dTAG^V^-1 for 24 h. Scale bar = 25 μm. (**C**) Quantification of EU incorporation in asynchronous cells and mitoses in (B). I = Interphase, M = Mitotic. Significance determined using paired Student's *t*-test. (**D**) Representative cell cycle profiles of asynchronous and prometaphase-arrested Parental HeLa and Gdown1 KO #3 HeLa cells. The average percentage of G2/M cells −/+ SEM is indicated at the top of each graph (*n* = 3 biological replicates). (**E**) Correlation of PRO-Seq pause region (top) and gene body (bottom) counts between asynchronous and mitotic Parental and Gdown1 KO #3 datasets (plotted data represent the sum of two replicates). (**F**) Boxplot analysis of pause region (top) and gene body (bottom) PRO-Seq counts in Parental and Gdown1 KO #3 mitotic cells. Significance was determined using a paired Student's t-test.

The observation that Gdown1 is primarily cytoplasmic in interphase cells and that its loss is associated with defective mitoses mirrors the localization and loss-of-function phenotype of TTF2, a Pol II termination factor that is involved in the eviction of all Pol II elongation complexes at the onset of mitosis. Importantly, Gdown1 potently inhibits TTF2 in vitro (18,57–59). At mitosis, redundant processes, including phosphorylation of general transcription factors (17), TTF2 function (18), and perhaps changes in chromatin accessibility (60,61), facilitate a global shutdown of transcription that is presumably required for the accurate deconvolution, condensation, and segregation of sister chromatids. We hypothesized that Gdown1 might also contribute to shut down or regulation of mitotic transcription by interfering with PIC assembly or by modulating, but not globally restricting, TTF2 function. As a first step, the localization of Gdown1 in mitotic cells was examined. In NHSFs and live eGFP-TEV-Gdown1 HAP1 cells Gdown1 appears to localize to the nucleus in early mitosis seemingly prior to complete breakdown of the nuclear envelope, similar to what is observed for TTF2 (57). Gdown1 apparently does not associate with mitotic chromatin in NHSFs, HAP1 cells, or HeLa cells, which mirrors the known distribution of Pol II in mitotic cells (16) (Figure 4A, Supplementary Figure S4A, B). Previous work suggested that Gdown1 is phosphorylated in mitotic cells at S270 (32), a finding that was further verified and extended by western blot analysis of samples derived from HeLa cells synchronized by and released from double thymidine block for up to 15 h or cells arrested at prometaphase of mitosis with Nocodazole (see methods for details of synchronization procedures). Gdown1 appears to undergo complete phosphorylation near the onset of mitosis, when Cyclin B1 levels peak, and its phosphorylation persists into the next G1 phase (Supplementary Figure S4C). Phosphorylation of Gdown1 modestly weakens its affinity for Pol II and its ability to inhibit TFIIF and TTF2 (32), and S270 falls within a described Pol II binding region (31). However, Pol II still forms potentially functional interactions with the phosphorylated form of Gdown1 in cells as it is recovered by Pol II IP (Supplementary Figure S4D). We hypothesize that phosphorylation of Gdown1 might increase its Pol II off-rate, thereby enabling TTF2 function and the efficient dissociation of Gdown1 from Pol II upon mitotic exit to facilitate its export from the forming early G1 nucleus.

Whether loss of Gdown1 was associated with an effect on mitotic transcription was next queried. EU incorporation assays were first employed, focusing on readily discernable metaphase structures which represent a phase when transcription is known to be shut down. Metaphase chromosomes displayed almost no EU staining in control cells (Figure 4B). Background-corrected measurements of EU incorporation in interphase and metaphase parental HeLa cells revealed only about a 5-fold reduction in incorporation on metaphases compared to interphase nuclei, a measurement that is affected by the background of EU signal across the cell (Figure 4C). As a positive control, when TTF2 was knocked down for 48 h (Supplementary Figure S4E) metaphase structures exhibited a significant increase in EU incorporation, reflecting the critical role of TTF2 in mitotic repression of transcription elongation (Figure 4B) (18). Gdown1 KO #3 HeLa cells did not exhibit a significant increase in EU incorporation on metaphases compared to metaphases in parental HeLa cells. Congruently, acute depletion of Gdown1 was not associated with a detectable increase in metaphase transcription compared to the DMSO control (Figure 4C). Although fluorescent EU incorporation assay is a reasonable approach to quantifying differences in mitotic transcription, its range of sensitivity is limited. This is particularly important in light of previous reports suggesting that mitotic transcription may be more profoundly inhibited (15,19,60,61). Further, it may be difficult to detect changes in transcription due to Gdown1 loss when redundant mechanisms of blocking transcription are still in operation.

To address the issue of sensitivity, spike-in controlled PRO-Seq was performed to directly ask whether mitotic transcription is affected in Gdown1 KO #3 HeLa cells. An established synchronization procedure (see methods) was utilized to harvest a sufficient number of HeLa cells synchronized at prometaphase for PRO-Seq. This protocol worked equally well at synchronizing parental and Gdown1 KO #3 HeLa cells, reproducibly giving >95% prometaphase cells (Figure 4D). Minor modifications to the nuclei isolation protocol were introduced to promote the retention of mitotic chromosomes (see methods), which were visualized via trypan blue staining of isolated mitotic ‘nuclei’ (Supplementary Figure S4F). Compared to asynchronous parental HeLa cells, prometaphase parental cells exhibited a significant, nearly 150-fold mean reduction in paused Pol II signal and 100-fold mean reduction in gene body transcription at 12 291 transcribed genes. Furthermore, it was apparent that the level of mitotic transcription strongly correlated with the level of transcription observed in asynchronous cells at the levels of pausing and productive elongation. By contrast, Gdown1 KO #3 cells exhibited a highly significant, but only ∼37-fold mean reduction paused Pol II signal and 17-fold mean reduction in gene Pol II PRO-Seq signal (Figure 4E, F and Supplementary Figure S4H). These observations were reproducible, as biologically duplicated PRO-Seq datasets exhibited a high level of correlation (Supplementary Figure S4G). Collectively, these data suggest that loss of Gdown1 may be associated with a partial de-repression of mitotic transcription, characterized by an increase in paused and productively elongating Pol II. However, we cannot exclude the possibility that these differences may be attributed to an indirect effect of Gdown1 knockout relating to abnormal cell cycle progression.

### Gdown1 modulates the combined functions of purified productive elongation factors

Our PRO-Seq analyses in mitotic cells indicated increased paused and productively elongating Pol II in Gdown1 KO #3 cells. At first glance, this is not wholly consistent with a model wherein Gdown1 inhibits transcription only at the level of initiation, as one might hypothesize that newly initiated elongating Pol II in mitotic cells would undergo rapid termination by TTF2. Indeed, single-molecular tracking studies suggest that Pol II exhibits a very short residence time on mitotic chromatin compared to residence times during interphase (61). Interestingly, a recent study reported that Gdown1 may regulate transcription at the level of productive elongation for a subset of genes in mouse liver, suggesting that Gdown1 association with elongating Pol II on these genes is somehow facilitative to transcription (36).

**Figure 5.**
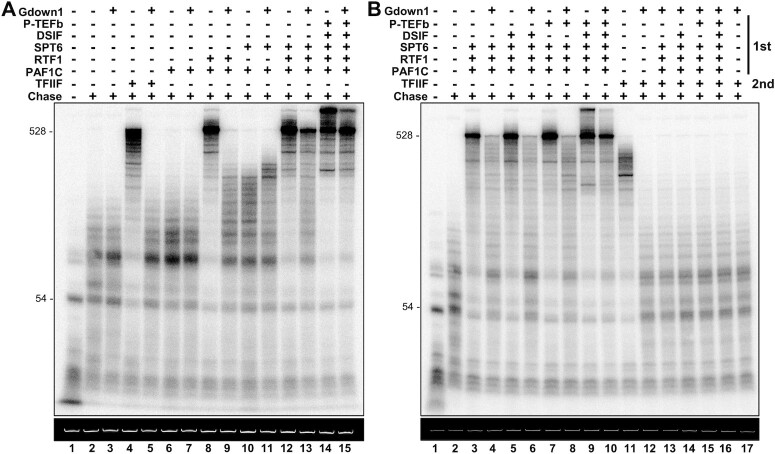
Gdown1 blocks the combined functions of purified productive elongation factors PAF1C, RTF1, SPT6, DSIF and P-TEFb. (**A**) Bulk isolated EECs were supplemented as indicated with 30 pmol of recombinant human Gdown1, incubated for 5 min, and then concentrated and washed to remove excess Gdown1. TFIIF (0.5 pmol) or productive elongation factors PAF1C (1 pmol), RTF1 (1 pmol), SPT6 (1 pmol), DSIF (0.3 pmol), and P-TEFb (0.5 pmol) were added to EECs or EEC(G)s. Samples were incubated for 10 min and then chased for 3 min, except for lane 1 in each panel which were not chased. The top autoradiograph shows labeled transcripts analyzed in a 6% TBE/urea gel. The bottom ethidium bromide stained gel shows the in vitro transcription template. (**B**) For lanes 1–10, as in (A), bulk isolated EECs were supplemented as indicated with 30 pmol of recombinant human Gdown1, incubated for 5 min, and then concentrated and washed to remove excess Gdown1. The above indicated amounts of productive elongation factors PAF1C, RTF1, SPT6, DSIF, and P-TEFb were added to EECs or EEC(G)s. Samples were incubated for 10 min and chased for 3 min then. For lanes 11–17, bulk isolated EECs were supplemented as indicated with 30 pmol of recombinant human Gdown1, incubated for 5 min, and then concentrated and washed to remove excess Gdown1. Indicated amounts of PE factors were added back and samples incubated for 10 min. Where P-TEFb was added, 500 μM ATP was included. EECs or EEC(G)s were then washed to remove PE factors, and 0.5 pmol of TFIIF was added back and incubated for 5 min before chasing the samples for 3 min. The autoradiograph shows labeled transcripts analyzed in a 6% TBE/urea gel, and then ethidium bromide stained gel is shown underneath.

The effect of Gdown1 on the activities of Pol II productive elongation (PE) factors has not yet been characterized in a defined in vitro system. To test this, recently purified PE factors PAF1C, RTF1, SPT6, DSIF, and P-TEFb (generously provided by Seychelle Vos and the Cramer lab) (2,41) were utilized to determine how their functions are impacted by Gdown1 in vitro. In these assays, Pol II early elongation complexes (EECs) were assembled on immobilized templates. Transcription was initiated with ^32^P-CTP and limiting NTPs and the resulting EECs were washed and resuspended in transcription buffer. A fraction of the EECs was combined with an excess of recombinant human Gdown1, forming EEC(G)s, and incubated for 5 min, after which remaining free Gdown1 was washed away. The indicated combinations of factors were added followed by 10 min incubations and 3 min of chase. Resulting transcripts were isolated and analyzed by TBE-urea PAGE (Figure 5A). As expected, Gdown1 had a slight positive effect on the elongation of otherwise naked EECs (lanes 2,3) (34). Further, Gdown1 robustly inhibited the elongation-stimulatory function of TFIIF, which has been previously documented (34,35) (lanes 4, 5). In agreement with prior findings, PAF1C alone (composed of PAF1, CTR9, LEO1 and CDC73, and WDR61) had a negligible impact on the rate of Pol II elongation, while the combination of PAF1C and RTF1 strongly stimulated Pol II elongation such that most Pol II ran off the template within 3 min (41). Strikingly, EECs containing Gdown1 were highly resistant to stimulation by PAF1C + RTF1 (lanes 6–9). The addition of RTF1 alone, which was assayed under two different experimental conditions, only slightly increased elongation rate, consistent with previous studies and reflecting its requirement for PAF1C (41) (Supplementary Figure S5). The combination of PAF1C and SPT6 modestly increased Pol II elongation rate, which curiously was further stimulated, albeit slightly, for EECs containing Gdown1 (Lanes 10,11). The combination of PAF1C, SPT6, and RTF1 strongly stimulated Pol II elongation rate. This stimulation was inhibited by Gdown1, yet to a lesser degree than for EECs only given PAF1C and RTF1 (lanes 12,13). Finally, the addition of PAF1C, SPT6, RTF1, DSIF and P-TEFb gave rise to a very high stimulation of Pol II elongation rate. These EECs were only modestly inhibited by Gdown1, suggesting that the combined functions of all tested PEs may be able to overcome inhibition by Gdown1 (lanes 14,15). Alternatively, this result may simply reflect the inability to accurately measure a decrease in elongation rate due to the high rates achieved through the additive effects of PE factors.

Identical and unique combinations of PEs were further tested in the same reaction format, yielding the reproducible conclusion that Gdown1 blocks elongation stimulation by a canonical set of Pol II PE factors (Figure 5B, lanes 1–10). To investigate further, it was tested whether transient exposure of static EECs containing Gdown1 to various combinations of PEs was sufficient to reverse Gdown1 inhibition and render the EECs susceptible to elongation stimulation by TFIIF (see methods). No combination of EECs alleviated Gdown1 inhibition of TFIIF (Figure 5B, lanes 1, 11–17). Of note, these results are consistent with our previous work showing that Gdown1 can remain functionally associated with productive elongation complexes reconstituted by the addition of nuclear extract to isolated elongation complexes and subsequently washed with high salt (34). Taken together, these in vitro transcription assays strongly suggest that Gdown1 blocks the combined functions of PE factors. However, Gdown1 may be modulated by PE factors in a manner that partially reverses Gdown1 inhibition of elongation stimulation and enables Gdown1 to remain associated with the productive elongation complex. Our findings have important implications for Gdown1 function during early mitotic transcription shutdown and in contexts where Gdown1 apparently behaves as a Pol II elongation factor (36).

## DISCUSSION

**Figure 6.**
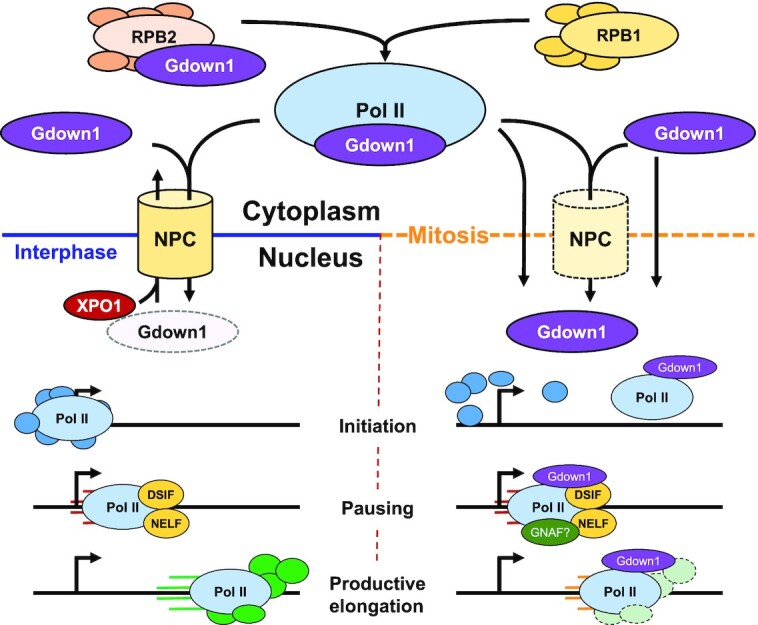
Model of Gdown1 regulation and function. During interphase (left), Gdown1 resides predominantly in the cytoplasm, where it may associate with the RPB2-containing Pol II sub-assembly and possibly the assembled polymerase, along with its biogenesis and import factors. Gdown1 shuttles into the nucleus either as a free protein or in association with Pol II. However, Gdown1 primarily resides in the cytoplasm due to XPO1/CRM1-dependent export. If low levels of Gdown1 associate with Pol II in the nucleus, competitive interactions with GTFs or Mediator function could remove Gdown1 from Pol II, enabling Gdown1 export. Transcription during interphase proceeds largely unaffected by Gdown1. At mitosis (right), modification of the nuclear pores and initial breakdown of the nuclear envelope enables cytoplasmic Gdown1 to enter the nucleus and associate with Pol II. Gdown1 may regulate transcription during mitosis through distinct mechanisms involving inhibition of PIC assembly, regulation of Pol II pausing, and modulation of productive elongation factor function.

Here, we report that the Pol II-interacting factor, Gdown1, affects transcription primarily during mitosis and is important for normal progression through the cell cycle. We found that Gdown1 is maintained in the cytoplasm during interphase by nuclear export and has essentially no direct effect on interphase transcription. It enters the nucleus at the onset of mitosis and exerts a transcriptionally repressive role at the level of initiation and/or productive elongation. As cells propagate in the absence of Gdown1, genome instability becomes evident, the p53 pathway is activated, and highly significant changes in gene expression occur that are distinct for each clonal line. A model that integrates what was learned about the cellular localization and function of Gdown1 during the cell cycle is presented in Figure 6.

Our study unambiguously establishes that Gdown1 is maintained in the cytoplasm of HeLa cells, HAP1 cells and NHSFs, at least in part, by nuclear export (Figure 6). These data sharply challenge the existing notion that Gdown1 is associated with a large fraction of Pol II (28). Although we found that all of the Gdown1 was associated with Pol II when cells were lysed with a detergent that disrupts the nuclear membrane, our results demonstrate that association of Gdown1 with Pol II occurs during the inevitable mixing of cytoplasmic Gdown1 with nuclear Pol II. Immunoprecipitation and mass spec analysis of Gdown1 demonstrated that the factor is associated with components of an RPB2/3-containing Pol II sub-assembly thought to be an intermediate in Pol II biogenesis along with the Pol II biogenesis and import factors RPAP2 and GPN3 (51,52,54–56). Additional support for Gdown1 interaction with cytoplasmic Pol II subassemblies comes from other studies which detected RPAP1, GPN1 and GPN2 associated with Gdown1 (29,62). The role of Gdown1 in Pol II assembly and nuclear import is still unclear because we did not observe any major defect in the import of Pol II in Gdown1-depleted cells. Several key pieces of data support the conclusion that Gdown1 does not play a major direct role in the control of transcription during interphase. Obviously, its cytoplasmic localization means that Gdown1 is largely unavailable to regulate transcription. Second, transient overexpression of wild-type Gdown1 did not impact transcription. Third, no significant differences in Pol II pausing, productive elongation, or enhancer transcription were detected following acute depletion of Gdown1 for 4 h, nor was there much occupancy of Gdown1 on chromatin. We did detect a very modest enrichment of HA-FKBP-Gdown1 near TSSs by ChIP-Seq, which is partially congruent with previous reports (29,34). However, our use of an unmodified cell line as a background control established that this occupancy is very low, which is expected given that Gdown1 is largely cytoplasmic. Since Gdown1 shuttles across the nuclear envelope during interphase, it is possible that our ChIP results reflect the association of the small amount of Gdown1 in the nucleus with Pol II transcription complexes. Alternatively, Gdown1 ChIP signals may derive predominantly from association of Gdown1 with Pol II in the minority of cells undergoing mitosis. Recent studies of Gdown1 in Drosophila embryos and mouse liver have suggested that Gdown1 can localize to the nucleus and directly regulate transcription (31,36,63). Thus, understanding the processes that control cellular localization of Gdown1 and its ability to interact with nuclear free or transcribing Pol II will be key to deciphering its transcription-regulatory roles across cell and tissue types.

Gdown1 is able to enter the nucleus at the onset of mitosis and contribute to the repression of mitotic transcription. This is reminiscent of TTF2, which is cytoplasmic until the onset of mitosis when it leads to termination of most engaged Pol II (18,59). Indeed, cytoplasmic sequestration during interphase seems to be an important control mechanism for other factors that carry out mitosis-restricted functions, such as Cyclin B1, Condensin I and PICH (64–66). We find that Gdown1 may carry out a transcription-repressive function during mitosis at the level of initiation, through established mechanisms involving inhibition of PIC assembly (29,31), and/or at the level of productive elongation (Figure 6). Inhibition of productive elongation factor function may promote Pol II termination, as this would likely slow Pol II, perhaps rendering it more susceptible to termination by TTF2. Of note, comparison of recently published structures of Pol II with Gdown1 (31) and the activated Pol II elongation complex containing RTF1 (41) suggest that Gdown1 may block the association of not only the LEO1 subunit of PAF1C, as noted by Jishage et al. (36), but also RTF1. This is consistent with the observation that Gdown1 robustly inhibits PAF1C and RTF1-dependent elongation stimulation in vitro. Previous in vitro studies of Gdown1 demonstrated that Gdown1 inhibits the combined functions of DSIF and NELF, but also cooperates with a partially purified negative accessory factor (GNAF) to facilitate pausing, adding further complexity to potential levels of Gdown1 transcriptional control during mitosis (34) (Figure 6).

Gdown1 knockout is associated with genome instability and gene expression changes that are largely inconsistent across clones. Apart from documented evidence of p53 pathway activation, which is likely a secondary effect of Gdown1 ablation, we detected only a modest overlap of gene expression changes among three knockout lines. These discrepancies may be attributed to genome instability leading to genetic variation driven by Gdown1 loss or to clonal variation in the parental HeLa cell line. Relating to other potential levels Gdown1 mitotic transcriptional control, several recent studies have investigated transcription reactivation upon mitotic exit and the roles of possible ‘bookmarking’ transcription factors and epigenetic marks that facilitate and specify transcription reactivation (15,61,67–70). That Gdown1 might influence transcription reactivation either globally or gene-specifically, through mechanisms involving modulation of initiation or PE factors, or interplay with the Mediator or TTF2, is an interesting possibility that perhaps warrants further investigation. Further, it is possible that Gdown1 may influence centromeric transcription, which has been suggested to occur during mitosis, and is important for genome integrity, centromere-kinetochore function, and the deposition of CENP-A in early G1 phase mammalian cells (71–73).

## DATA AVAILABILITY

All raw and processed sequencing data are made available under NCBI GEO Accession Number GSE186133. Previously published H3K4me1 and H3K4me3 ChIP-Seq data that were utilized in this study are available at NCBI GEO Accession Number GSE100742. Previously published HeLa NasCap data that were utilized in this study are available at NCBI GEO Accession Number GSE139237.

## Supplementary Material

gkac015_Supplemental_Files
