# Supplementary material for: Nuclear export restricts Gdown1 to a mitotic function
Source: Nucleic Acids Res. 2022 Jan 20;50(4):1908–26. doi: 10.1093/nar/gkac015 (PMC8887472; doi:10.1093/nar/gkac015)
Supplement: gkac015_Supplemental_Files [file gkac015_supplemental_files.zip › Ball et al. Supplementary Figures.pdf]

Nuclear export restricts Gdown1 to a mitotic function

Christopher B. Ball, Mrutyunjaya Parida, Juan F. Santana, Benjamin M. Spector, Gustavo A. Suarez, and David H. Price

Department of Biochemistry and Molecular Biology, The University of Iowa, Iowa City, IA 52242, USA

Corresponding author: David Price (david-price@uiowa.edu)

Supplementary Figures

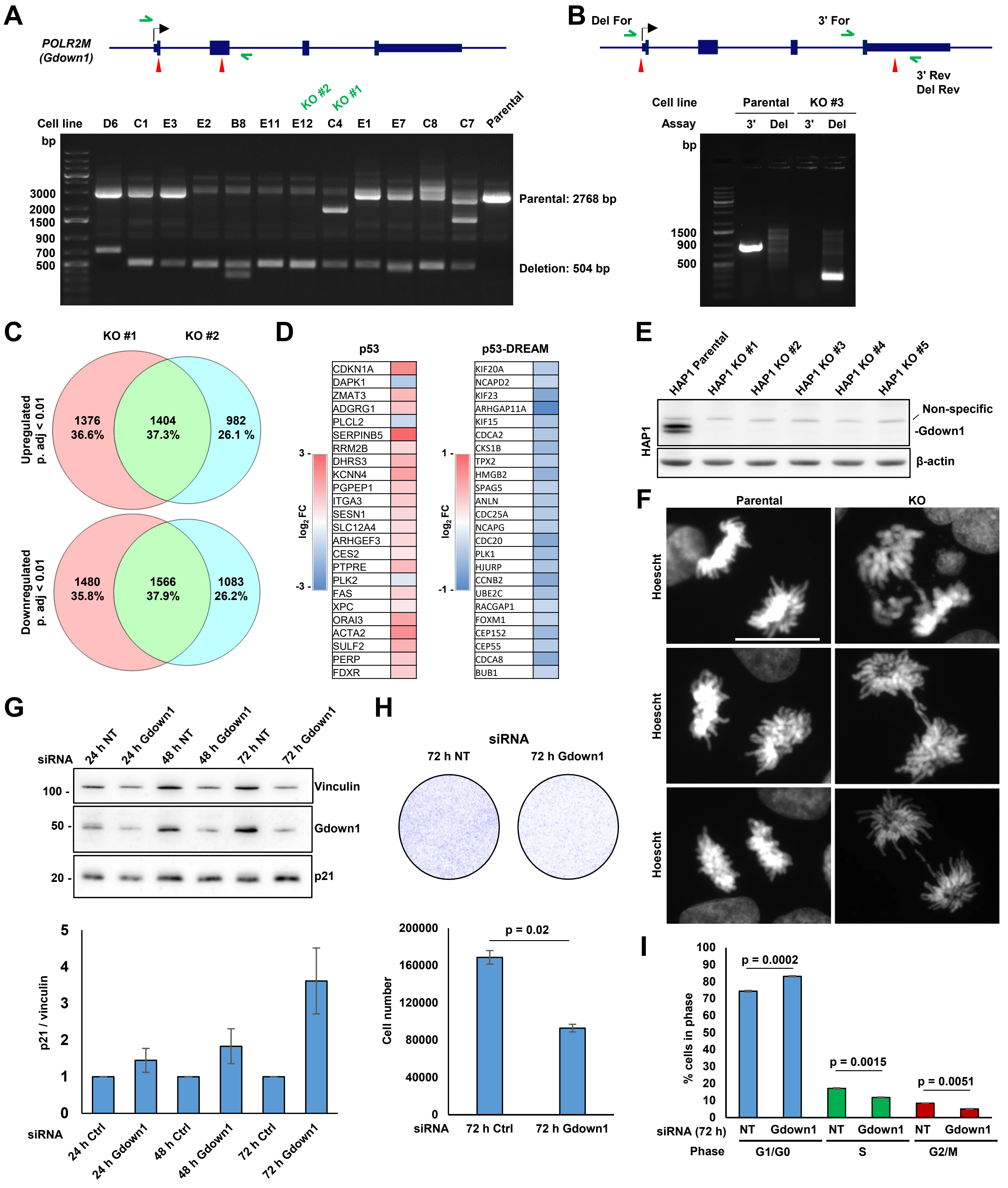
A
POLR2M (Gdown1)
Cell line
D6 C1 E3 E2 B8 E11 E12 C4 E1 E7 C8 C7 Parental
KO #2 KO #1
bp
3000
2000
1500
900
700
500
Parental: 2768 bp
Deletion: 504 bp
B
Del For
3' For
3' Rev
Del Rev
Cell line
Parental KO #3
Assay
3' Del 3' Del
bp
1500
900
500
C
KO #1 KO #2
Upregulated p. adj < 0.01
1376 36.6%
1404 37.3%
982 26.1 %
Downregulated p. adj < 0.01
1480 35.8%
1566 37.9%
1083 26.2%
D
p53
CDKN1A
DAPK1
ZMAT3
ADGRG1
PLCL2
SERPINB5
RRM2B
DHRS3
KCNN4
PGPEP1
ITGA3
SESN1
SLC12A4
ARHGEF3
CES2
PTPRE
PLK2
FAS
XPC
ORAI3
ACTA2
SULF2
PERP
FDXR
p53-DREAM
KIF20A
NCAPD2
KIF23
ARHGAP11A
KIF15
CDCA2
CKS1B
TPX2
HMGB2
SPAG5
ANLN
CDC25A
NCAPG
CDC20
PLK1
HJURP
CCNB2
UBE2C
RACGAP1
FOXM1
CEP152
CEP55
CDCA8
BUB1
log2 FC
3
-3
1
-1
E
HAP1 Parental
HAP1 KO #1
HAP1 KO #2
HAP1 KO #3
HAP1 KO #4
HAP1 KO #5
HAP1
Non-specific
Gdown1
β-actin
F
Parental
KO
Hoescht
Hoescht
Hoescht
G
siRNA
24 h NT
24 h Gdown1
48 h NT
48 h Gdown1
72 h NT
72 h Gdown1
100 -
50 -
20 -
Vinculin
Gdown1
p21
p21 / vinculin
5
4
3
2
1
0
siRNA
24 h Ctrl
24 h Gdown1
48 h Ctrl
48 h Gdown1
72 h Ctrl
72 h Gdown1
H
siRNA
72 h NT
72 h Gdown1
Cell number
200000
160000
120000
80000
40000
0
p = 0.02
siRNA
72 h Ctrl
72 h Gdown1
I
% cells in phase
100
90
80
70
60
50
40
30
20
10
0
p = 0.0002
p = 0.0015
p = 0.0051
siRNA (72 h)
NT Gdown1 NT Gdown1 NT Gdown1
Phase
G1/G0
S
G2/M

Supplementary Figure S1. (A) Top: Schematic of the Gdown1 locus, indicating Cas9 target sites for development of Gdown1 KO lines #1 and #2 (red arrows), and the approximate locations of primers used to assay for deletion (single-headed green arrows). Bottom: Representative screen of Gdown1 KO clones for target region deletion of PCR and TAE agarose gel electrophoresis. Clones are alphanumerically named by their location within a 96-well plate. Expected sizes of the full-length/wild-type and deletion PCR products are indicated at the bottom of the gel. Clones that were further analyzed by western blot and confirmed Gdown1 knockouts are named KO #1 and #2 in green. Note that Gdown1 KO line #1 is apparently heterozygous, but no wild-type PCR product is detected, nor is any Gdown1 protein by Western blot (Figure 1A). (B) Top: Schematic of the Gdown1 locus, indicating Cas9 target sites for development of Gdown1 KO line #3 (red arrows). Two PCR assays that utilized three primers (indicated by single-headed green arrows) were employed to assay for deletion, as PCR could not amplify across the entire gene. The Del For primer in combination with the 3' Rev primer yields a product only when the intervening region targeted for deletion is removed. The 3' For primer in combination with the 3' Rev primer only yields a product when the gene is intact, as the forward primer falls within the region targeted for deletion. Bottom: Confirmation of knockout in Gdown1 KO #3 cells by PCR and TAE agarose gel electrophoresis. (C) Venn diagram analysis of overlap of differentially upregulated (top) and downregulated (bottom) genes (p. adj. < 0.05) between Gdown1 KO #1 and #2 cells relative to the parental cell line based on analysis of total RNA-Seq data. (D) Log_2 fold-change in expression of genes transactivated by p53 and indirectly repressed by the p53-DREAM pathway. Shown are top 25 most significant genes in each set ranked by increasing adjusted p-value (for all, $p < 0.01$). (E) Western blot analysis of Gdown1 expression in Parental HAP1 cells and 5 separate HAP1 Gdown1 KO clones in which the entire Gdown1 CDS was targeted for deletion. (F) Hoescht staining of Parental and Gdown1 KO HAP1 cells exhibiting anaphases. Scale bar = 25 µm. (G) Top: Western blot analysis of Vinculin (loading control), Gdown1, and p21 expression in NHSF cells at 24, 48, or 72 hours following transfection of non-targeting (NT) or Gdown1 mRNA-targeting siRNAs. Bottom: Quantification of p21 levels relative the vinculin loading control at each time point. Quantification was carried out in ImageJ (n = 3 biological replicates). Data are presented as Mean ± SEM. (H) Top: Representative scans of crystal violet stained NHSFs transfected with non-targeting or Gdown1 mRNA-targeting siRNA for 72 h. Bottom: Quantification of cell biomass. Data are presented as Mean ± SEM. Significance was determined using a paired Student's *t*-test). (I) Quantification of cell-cycle distribution in NHSFs treated with non-targeting or Gdown1 mRNA-targeting siRNAs for 72 h. Data are presented as Mean ± SEM. Significance was determined using a paired Student's *t*-test).

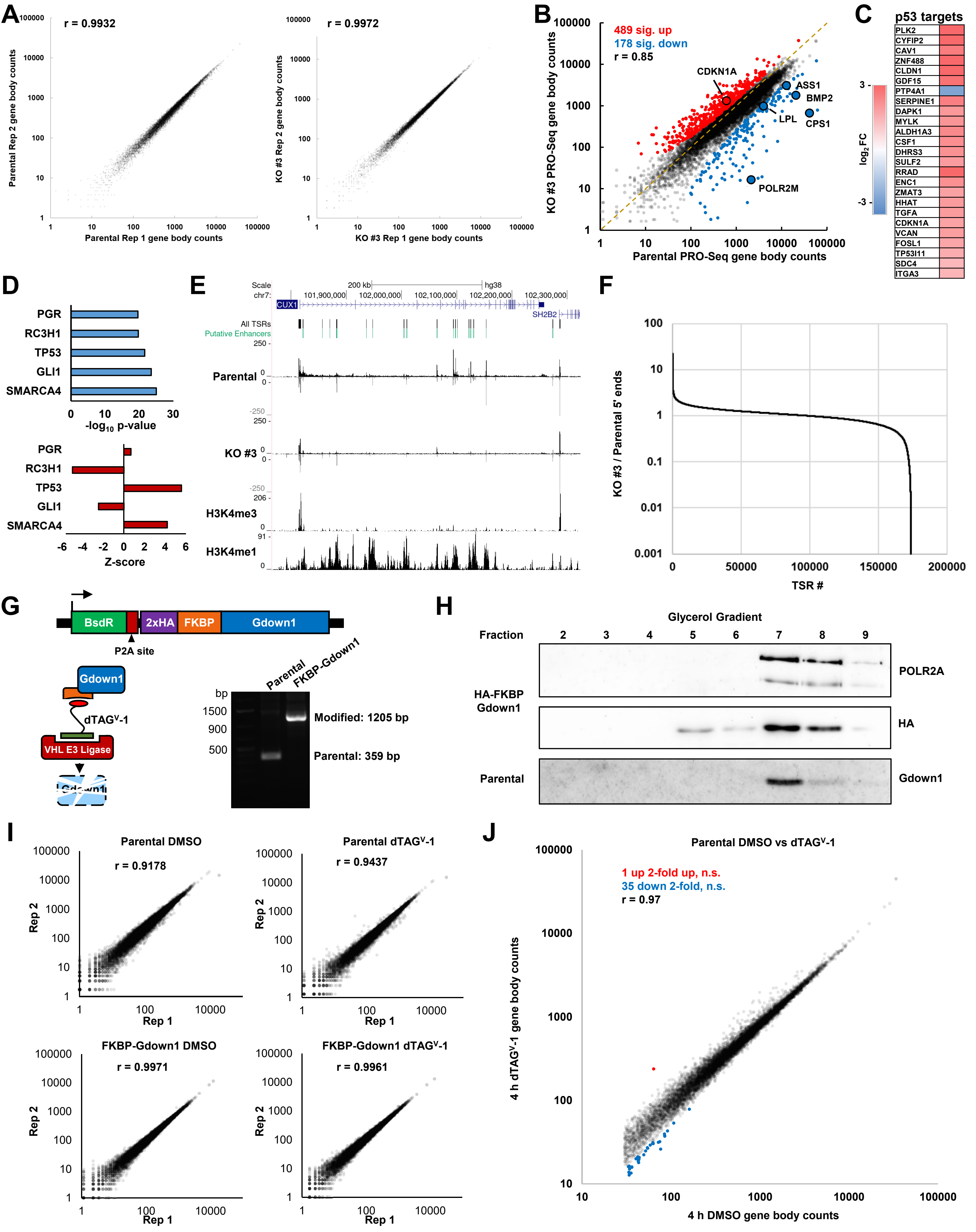

A
r = 0.9932
Parental Rep 2 gene body counts
Parental Rep 1 gene body counts
r = 0.9972
KO #3 Rep 2 gene body counts
KO #3 Rep 1 gene body counts
B
489 sig. up
178 sig. down
r = 0.85
CDKN1A
ASS1
BMP2
LPL
CPS1
POLR2M
KO #3 PRO-Seq gene body counts
Parental PRO-Seq gene body counts
C
p53 targets
PLK2
CYFIP2
CAV1
ZNF488
CLDN1
GDF15
PTP4A1
SERPINE1
DAPK1
MYLK
ALDH1A3
CSF1
DHRS3
SULF2
RRAD
ENC1
ZMAT3
HHAT
TGFA
CDKN1A
VCAN
FOSL1
TP53I11
SDC4
ITGA3
log2 FC
D
PGR
RC3H1
TP53
GLI1
SMARCA4
-log10 p-value
Z-score
E
Scale
chr7:
200 kb
hg38
101,900,000
102,000,000
102,100,000
102,200,000
102,300,000
CUX1
SH2B2
All TSRs
Putative Enhancers
Parental
KO #3
H3K4me3
H3K4me1
F
KO #3 / Parental 5' ends
TSR #
G
BsdR
2xHA
FKBP
Gdown1
P2A site
dTAGV-1
VHL E3 Ligase
Parental
FKBP-Gdown1
bp
1500
900
500
Modified: 1205 bp
Parental: 359 bp
H
Glycerol Gradient
Fraction
HA-FKBP Gdown1
Parental
POLR2A
HA
Gdown1
I
Parental DMSO
r = 0.9178
Parental dTAGV-1
r = 0.9437
FKBP-Gdown1 DMSO
r = 0.9971
FKBP-Gdown1 dTAGV-1
r = 0.9961
Rep 1
Rep 2
J
Parental DMSO vs dTAGV-1
1 up 2-fold up, n.s.
35 down 2-fold, n.s.
r = 0.97
4 h dTAGV-1 gene body counts
4 h DMSO gene body counts

Supplemental Figure S2. (A) Correlation analyses of Parental and Gdown1 KO #3 PRO-Seq duplicates. r = Pearson's correlation coefficient. (B) Correlation of Parental vs. Gdown1 KO #3 PRO-Seq gene body counts. Genes that exhibit a significant (p. adj. < 0.01) change in transcription are colored blue or red for downregulated or upregulated in KO #3 cells, respectively. Specifically referenced genes in the manuscript are indicated. N = 12,291 genes. (C) Top 25 differentially transcribed p53 target genes (Gdown1 KO #3 vs Parental, p. adj <0.01). Fold changes are indicated by the colored scale. (D) Top 5 upstream regulators of differentially transcribed genes as identified by Qiagen Ingenuity Platform Analysis. Top: p-value of gene set overlaps. Bottom: Activation Z-scores. (E) UCSC genome browser snap shot showing Parental and Gdown1 KO #3 PRO-Seq, and Parental H3K4me3 and H3K4me1 coverage of the CUX1 gene, which contains several intragenic, bioinformatically defined putative enhancers that are marked by islands of H3K4me1 signal and are less transcribed in Gdown1 KO #3 cells. The top tracks indicate all identified TSRs in black the subset defined as putative enhancers according to defined parameters (see methods) in green. (F) 20 bp TSRs sorted by ratio of Gdown1 KO #3 / Parental 5' end counts. N = 174,098 TSRs. (G)Top: Schematic of the endogenous modification introduced at the Gdown1 locus, which adds sequence encoding a Blasticidin resistance gene, P2A ribosomal skipping sequence, 2 tandem HA tags, and an FKBP degron domain to the Gdown1 N-terminus. Bottom left: Schematic for induced proteolytic degradation of HA-FKBP-Gdown1 by dTAG^{V}-1. Bottom right: PCR verification of desired knock-in at the Gdown1 N-terminus. Expected product sizes are indicated at the bottom of the gel. (H) Glycerol gradient fractionation of cell lysates from Parental and HA-FKBP-Gdown1 HeLa cells. HA-FKBP-Gdown1 peaks in the same fraction as wild-type Gdown1 and co-fractionates almost entirely with free POLR2A. (I) Correlation analyses of PRO-Seq duplicates (counts over gene body) for Parental and HA-FKBP Gdown1 DMSO and dTAG^{V}-1 treatments. r = Pearson's correlation coefficient. (J) Correlation of Parental DMSO vs. VHL PRO-Seq data. r = Pearson's correlation coefficient. No significant differences (p adj. < 0.05) in transcription were detected through pairwise comparison of gene body counts with DESeq2.

A

Immunoprecipitated proteins identified by LC-MS				
**Protein**	**Unique peptides**	**ID probability**	**Description**	**Cellular localization**
POLR2M	6	100%	Gdown1 / Immunoprecipitated protein	Primarily cytoplasmic
**POLR2B**	8	100%	Pol II subunit	Nuclear
POLR2C	5	100%	Pol II subunit	Nuclear
POLR2I	1	98%	Pol II subunit	Nuclear
POLR2L	1	98%	Pol I / II / III subunit	Nuclear
RPAP2	1	98%	Pol II biogensis / import factor	Cytoplasmic and nuclear
GPN3	2	100%	Pol II biogensis / import factor	Cytoplasmic and nuclear
**POLR2A**	7	100%	Pol II subunit	Nuclear
POLR2G	1	98%	Pol II subunit	Nuclear

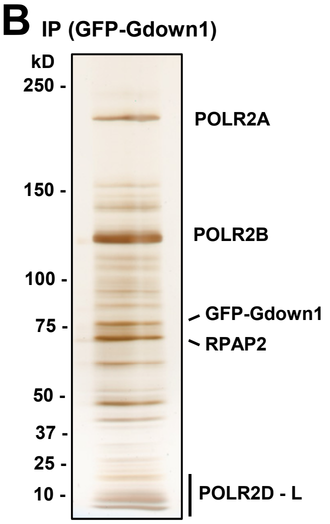

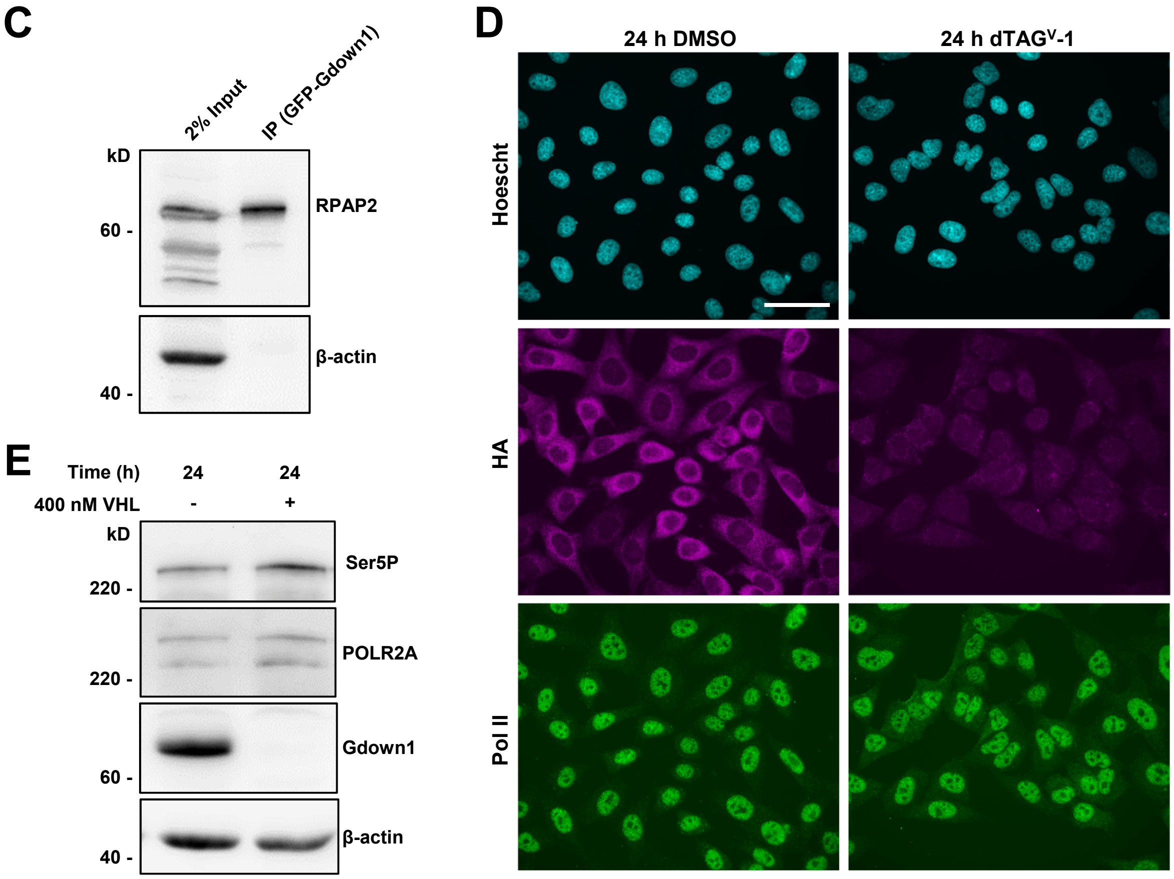

Supplemental Figure S3. (A) Summary of mass spec data identifying proteins immunopecipitated with GFP-Gdown1. Proteins are grouped by their presence in one of two cytoplasmic Pol II sub-assemblies, sub-assembly I (containing POLR2B and POLR2C), and sub-assembly II (containing POLR2A). (B) Silver stained gel showing proteins immunoprecipitated with GFP-Gdown1. Major species are assigned identifications. (C) Western blot showing immunoprecipitation of RPAP2 by GFP-Gdown1. (D) Indirect immunofluorescence for HA-FKBP-Gdown1 and Pol II in HA-FKBP-Gdown1 cells treated for 24 h with DMSO or dTAG^{V}-1. Scale bar = 50 µm. (E) Western blot showing levels of Gdown1, POLR2A, and SerP Pol II in HA-FKBP-Gdown1 cells treated for 24 h with DMSO or dTAG^{V}-1.

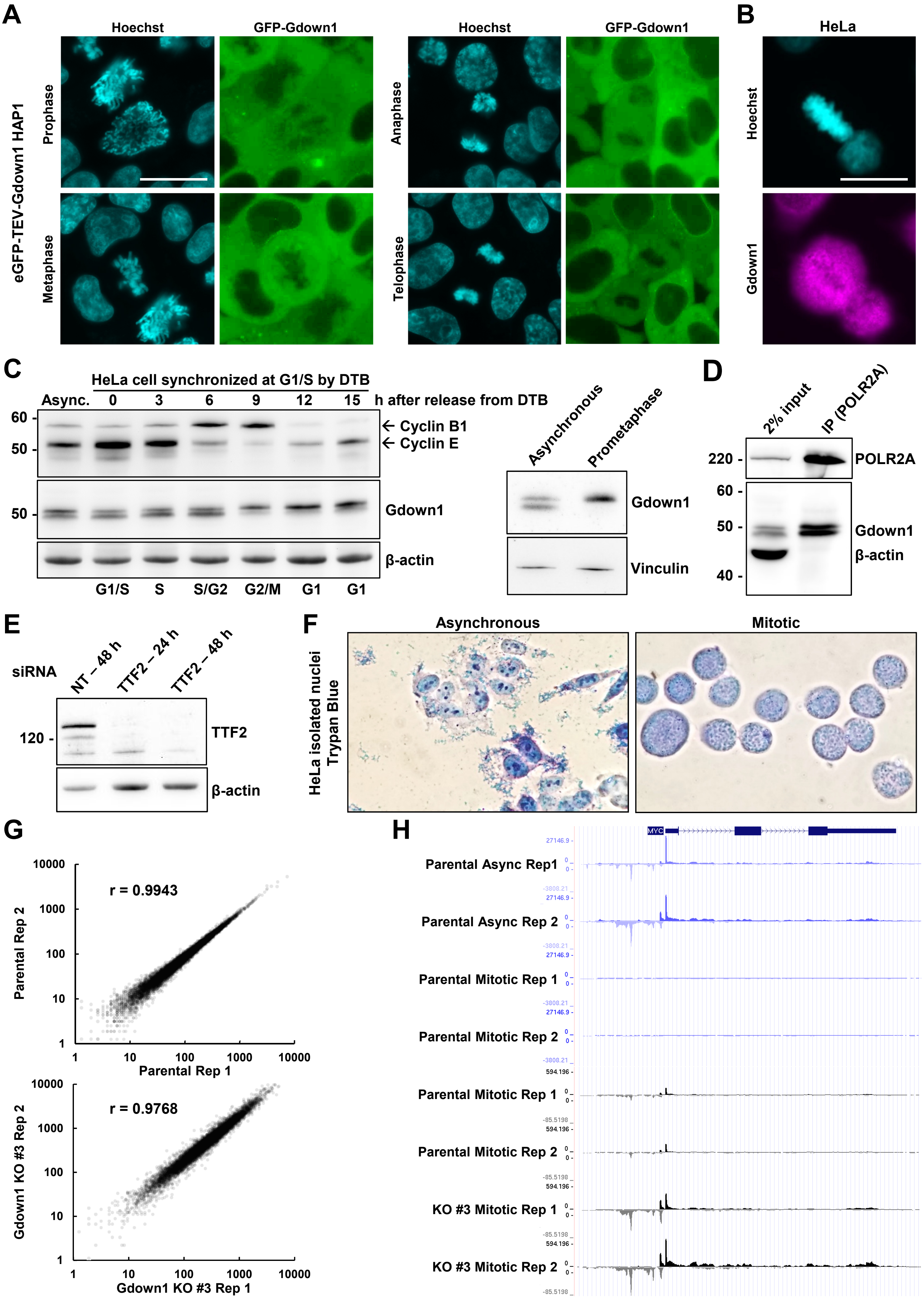
A
Hoechst
GFP-Gdown1
eGFP-TEV-Gdown1 HAP1
Prophase
Metaphase
Anaphase
Telophase
B
HeLa
Hoechst
Gdown1
C
HeLa cell synchronized at G1/S by DTB
Async.
0
3
6
9
12
15
h after release from DTB
60
50
← Cyclin B1
← Cyclin E
Gdown1
β-actin
G1/S
S
S/G2
G2/M
G1
G1
Asynchronous
Prometaphase
Gdown1
Vinculin
D
2% input
IP (POLR2A)
220
POLR2A
60
50
40
Gdown1
β-actin
E
siRNA
NT – 48 h
TTF2 – 24 h
TTF2 – 48 h
120
TTF2
β-actin
F
Asynchronous
Mitotic
HeLa isolated nuclei
Trypan Blue
G
r = 0.9943
Parental Rep 2
Parental Rep 1
r = 0.9768
Gdown1 KO #3 Rep 2
Gdown1 KO #3 Rep 1
1
10
100
1000
10000
H
MYC
Parental Async Rep1
Parental Async Rep 2
Parental Mitotic Rep 1
Parental Mitotic Rep 2
Parental Mitotic Rep 1
Parental Mitotic Rep 2
KO #3 Mitotic Rep 1
KO #3 Mitotic Rep 2
27146.9
-3808.21
594.196
-85.5198
0

Supplementary Figure S4. (A) Visualization of eGFP-TEV-Gdown1 in live HAP1 cells in all mitotic phases. Scale bar = 25 µm. (B) Indirect immunofluorescence detecting Gdown1 in a metaphase HeLa cell. Scale bar = 25 µm. (C) Left: Western blot analysis of Cyclin B1, Cyclin E, Gdown1, and β-actin in asynchronous HeLa cells or HeLa synchronized to G1/S by double-thymidine block, released, and harvested every 3 h starting at t = 0 h for 15 h. Cyclin E exhibits peak expression at the onset of S phase, and Cyclin B1 rises as cells enter G2, peaks at G2/M, and is rapidly depleted upon mitotic exit. Approximate phases are indicated at the bottom of the gel. Right: Western blot analysis of Vinculin and Gdown1 in asynchronous HeLa cells and HeLa cells arrested at prometaphase. (D) Western blot showing immunoprecipitation of unphosphorylated and phosphorylated Gdown1 by western blot. (E) Western blot showing TTF2 levels following 24 and 48 h transfection with non-targeting (NT) or TTF2 mRNA-targeting siRNAs. (F) Representative phase-contrast images of Trypan blue-stained asynchronous nuclei and mitotic 'nuclei' containing condensed chromosomes harvested for PRO-Seq. (G) Correlation of gene body counts between mitotic PRO-Seq duplicates in Parental and Gdown1 KO #3 HeLa cells. r = Pearson's correlation coefficient. (H) UCSC genome browser snapshots showing transcriptional downregulation of the Myc gene in mitotic cells compared to asynchronous cells (top), and increase in mitotic transcription in Gdown1 KO #3 cells compared to mitotic transcription in Parental HeLa cells (bottom).

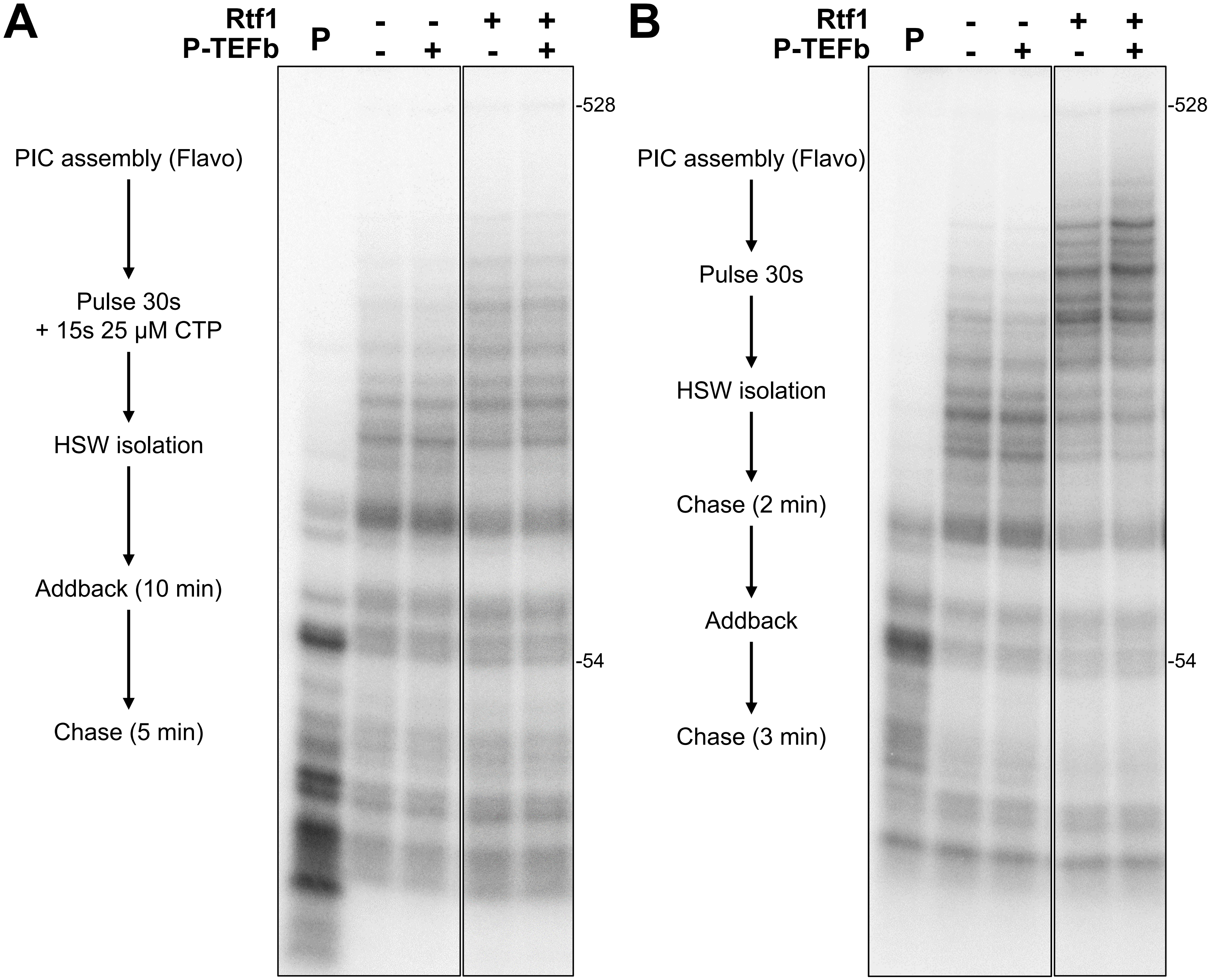

A
Rtf1
P-TEFb
P
- - + +
- + - +
-528
-54
PIC assembly (Flavo)
Pulse 30s
+ 15s 25 µM CTP
HSW isolation
Addback (10 min)
Chase (5 min)
B
Rtf1
P-TEFb
P
- - + +
- + - +
-528
-54
PIC assembly (Flavo)
Pulse 30s
HSW isolation
Chase (2 min)
Addback
Chase (3 min)

Supplementary Figure S5. (A) In vitro transcription assay showing RTF1-dependent stimulation of transcription elongation. PICs were assembled on an immobilized template in the presence of Flavopiridol to block P-TEFb activity during the pulse. Complexes were pulsed-labeled for 30 s, followed by extension for 15 s in the presence of 25 μM cold CTP. ECs were isolated, the indicated factors were added back and incubated for 10 minutes, and the ECs were chased for 5 minutes. RNA was isolated and analyzed by TBE Urea-PAGE. (B) An in vitro transcription assay similar to (A), with the noted modifications to the pulse, chase, and addback strategies shown in the assay schematic.
