# Supplementary material for: Nuclear export restricts Gdown1 to a mitotic function
Source: Nucleic Acids Res. 2022 Jan 20;50(4):1908–26. doi: 10.1093/nar/gkac015 (PMC8887472; doi:10.1093/nar/gkac015)
Supplement: gkac015_Supplemental_Files [file gkac015_supplemental_files.zip › Supplementary Data File legend.docx]

**Supplementary Data File**

This Excel file contains 7 sheets that provide additional information about the experiments performed and details about the analysis of results. Antibodies contains the identity, source, catalog number, and dilutions used for westerns and immunofluorescence. Nucleic Acid Reagents lists all oligos used for PCRs and CRISPR experiments. KO#1 and 2 vs. Parental RNA-Seq is a table that lists genes and their changes including statistics. PRO-Seq Normalization Factors lists the Spike-in correction factors for all PRO-Seq datasets. DESeq2 Parental vs. KO#3 GB is a table of gene body quantification. truQuant HA-FKBB-Gdown1 GB is a table that provides quantification of the PRO-Seq counts before and after depletion of Gdown1. Putative Enhancers gives the location of enhancers defined by the ratio of H3K4me1 to H3K4me3.
